# The application of emerging immunotherapy in the treatment of prostate cancer: progress, dilemma and promise

**DOI:** 10.3389/fimmu.2025.1544882

**Published:** 2025-03-12

**Authors:** Jizhong Che, Yuanyuan Liu, Yangyang Liu, Jingheng Song, Hongguo Cui, Dongdong Feng, Aimin Tian, Zhengchao Zhang, Yankai Xu

**Affiliations:** ^1^ Department of Urology, Yantai Affiliated Hospital of Binzhou Medical University, The Second Clinical Medical College of Binzhou Medical University, Yantai, Shandong, China; ^2^ Department of Urology, Haiyang City People’s Hospital, Yantai, Shandong, China

**Keywords:** prostate cancer, immunotherapy, TMEs, cancer vaccines, immune checkpoint inhibitors, adoptive cell therapy, CAR-T cells

## Abstract

In recent years, there has been a growing trend towards the utilization of immunotherapy techniques for the treatment of cancer. Some malignancies have acquired significant progress with the use of cancer vaccines, immune checkpoint inhibitors, and adoptive cells therapy. Scholars are exploring the aforementioned methods as potential treatments for advanced prostate cancer (PCa) due to the absence of effective adjuvant therapy to improve the prognosis of metastatic castration-resistant prostate cancer (mCRPC). Immunotherapy strategies have yet to achieve significant advancements in the treatment of PCa, largely attributed to the inhibitory tumor microenvironment and low mutation load characteristic of this malignancy. Hence, researchers endeavor to address these challenges by optimizing the design and efficacy of immunotherapy approaches, as well as integrating them with other therapeutic modalities. To date, studies have also shown potential clinical benefits. This comprehensive review analyzed the utilization of immunotherapy techniques in the treatment of PCa, assessing their advantages and obstacles, with the aim of providing healthcare professionals and scholars with a comprehensive understanding of the progress in this field.

## 1 Introduction

Among elderly males, prostate cancer (PCa) is a prevalent form of cancer, coming in second in terms of occurrence globally among male malignancies and ranking as the fifth leading cause of death worldwide (1). In 2020, the number of new PCa cases worldwide has exceeded 1.4 million (2). For the clinical treatment of organ-localized PCa, radical prostatectomy combined with chemoradiotherapy and other therapeutic strategies are commonly used, and the therapeutic effect is excellent, and majority patients can even be cured (3). Androgen deprivation therapy (ADT) is the primary treatment for locally advanced and metastatic PCa. Although testosterone levels decrease post-treatment, many patients eventually develop resistance to the drugs and advance to castration-resistant prostate cancer (CRPC) (4). A portion of these individuals will eventually develop metastatic castration-resistant prostate cancer (mCRPC), with a 5-year survival rate of less than 30% for these patients (5).

Currently, there is rapid progress in research on immunotherapy as an adjuvant therapy for the treatment of mCRPC. Scholars endeavored to utilize immunotherapy, specifically immune checkpoint inhibitors (ICIs), tumor vaccines, and chimeric antigen receptor T cells (CAR-T), in the treatment of patients diagnosed with mCRPC. Currently, the majority of clinical studies have demonstrated that the aforementioned strategies exhibit favorable safety profiles, with certain studies also indicating notable clinical effectiveness. The FDA has authorized sipuleucel-T (targeting prostate acid phosphatase) as the initial immunotherapy treatment for asymptomatic or mild symptomatic mCRPC (5, 6). Furthermore, the researchers attempted to enhance patient prognosis by employing immunotherapy strategies in conjunction with chemoradiotherapy and other interventions, resulting in promising initial outcomes (7). This review summarizes the immunosuppression mechanism of PCa, and comprehensively summarizes the application of immunotherapy in PCa treatment in recent years, and discusses its existing value and difficulties, aiming at providing convenience for relevant clinicians and researchers to understand the progress in this field.

## 2 Immunosuppressive mechanism of PCa

**Figure 1:**
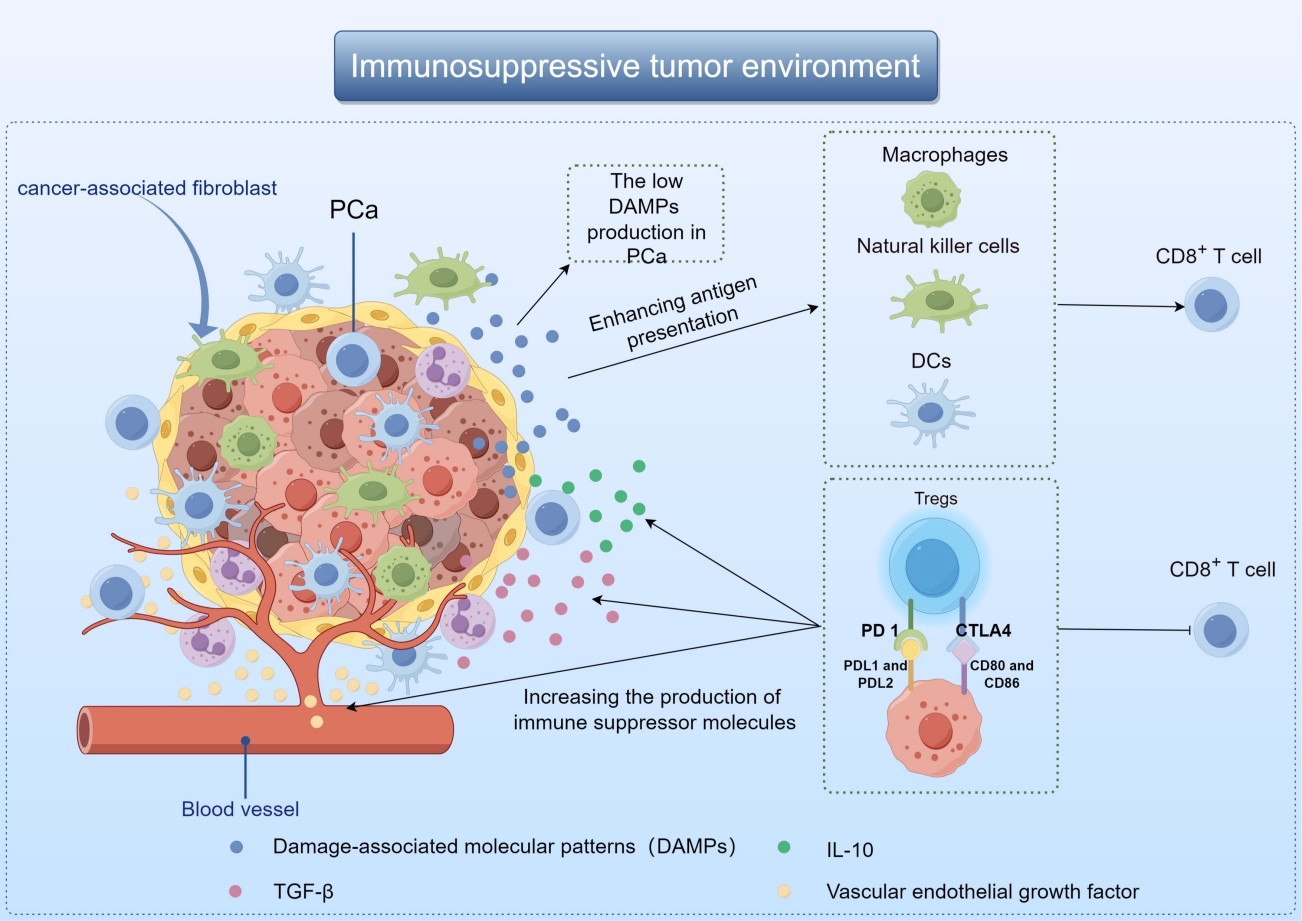
Demonstration of immunosuppressive mechanisms in the TME of PCa by Figdraw.

The poor immunotherapy response of PCa may be related to TME. Composed of immune cells, blood vessels and extracellular matrix components, TME plays the most important role in tumor progression and therapeutic response (8). It influences tumor behavior through interaction with surrounding macromolecules and tissues, such as inhibiting or stimulating tumor growth (9, 10). When tumor cells die, they release damage-associated molecular patterns (DAMPs) that activate macrophages, dendritic cells (DCs), and natural killer cells. These immune cells then release anti-tumor cytokines, and promote DCs maturation and antigen presentation (11, 12). The low DAMPs production in PCa results in the affected activation of DCs, which in turn reduces the production of immune-related chemokines and cytokines, and ultimately leads to the loss of T cells around the tumor (13). Besides, PCa TME also promotes the growth of immunosuppressive cells, especially regulatory T cells (Tregs). Cytotoxic T lymphocyt-associated protein 4 (CTLA4), programmed death receptor 1 (PD-1) and other inhibitory molecules were present on the cell membrane of Tregs. These compounds have the ability to block T cell activity, increase the production of immune suppressor molecules like transforming growth factor-β(TGF-β), vascular endothelial growth factor, and interleukin-10(IL-10), leading to the eventual spread of cancer cells (**Figure 1**) (14, 15).

Some scholars also believe that the poor effect of PCa immunotherapy may be related to the low tumor mutation burden (TMB). TMB is the mutation rate of genes in each coding region of tumor genome, and the mutation rate is different in different tumors. PCa has a mutation rate of about 1 individual cell mutation per megabase, while lung cancer or melanoma has 10 or more somatic mutations per megabase (16). Simultaneously, the emergence of neoantigens is associated with diverse types of gene mutations, with tumors exhibiting a higher somatic mutation burden producing a greater quantity of neoantigens (17). Research has indicated that certain PCa patients exhibit an elevated mutation rate in genes associated with DNA damage repair, resulting in a higher TMB and consequently a more favorable response to immunotherapy (18–20). It can be seen that the efficacy of immunotherapy is related to TMB, and most PCa patients have low TMB, resulting in poor efficacy of PCa immunotherapy (21). The application of immunotherapy to the treatment of PCa needs to overcome the resistance associated with low TMB.

In the context of immunotherapy for PCa, another significant challenge that must be addressed is the phenomenon of tumor immune escape, a common obstacle encountered in various forms of cancer treatment. Tumor immune evasion primarily encompasses two facets: Firstly, tumor cells evade immune detection through mechanisms such as diminished immune responses to tumor antigens. Additionally, alterations in the body’s immune system function, including the inability to detect low levels of tumor-associated antigens (TAAs) in the early stages of tumor development and the ineffectiveness of antigen-presenting cells (APCs) (22). Current studies have shown that many types of tumors show good clinical response to immunotherapy (23–25). Among them, cancer vaccines, ICIs, and CAR-T cells are demonstrating promising outcomes. The aforementioned treatment modalities for PCa have demonstrated only preliminary efficacy, necessitating further advancement before widespread clinical implementation.

## 3 Cancer vaccines are used to treat PCa

### 3.1 Cancer vaccines

**Figure 2:**
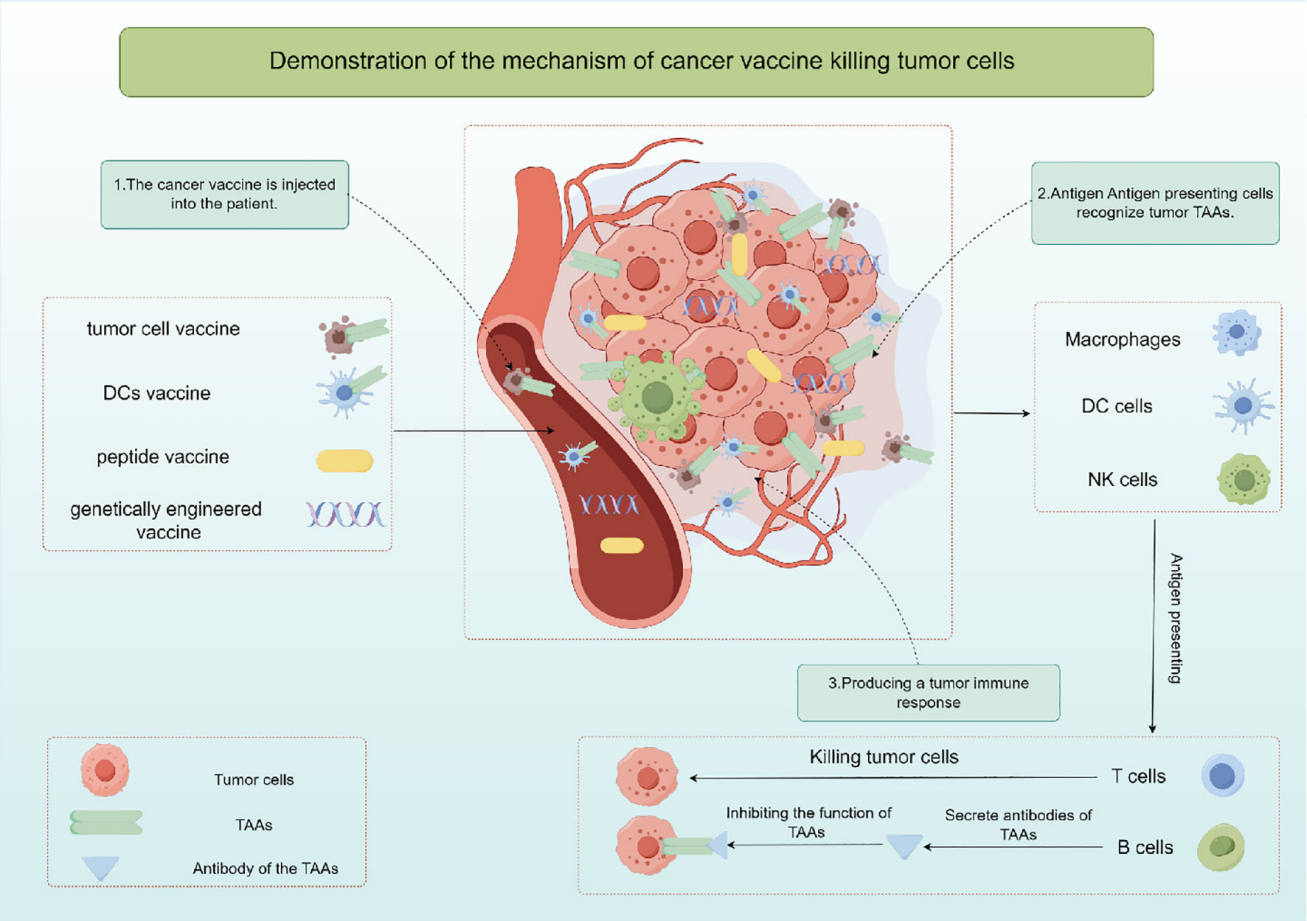
The mechanism of action and classification of cancer vaccines by Figdraw.

The cancer vaccine is a kind of active immunotherapy, which induces specific immune response to produce tumor killing effect by exogenous injection of human TAAs (26). It is expected to provide effective strategies for cancer prevention and treatment, and includes autologous tumor cell vaccine, DCs vaccine, peptide vaccine and genetically engineered vaccine according to the different components injected into the body. Cancer vaccine primarily works by stimulating T cells, B cells, and other immune cells using specific TAAs, resulting in the elimination of cancerous cells (**Figure 2**). Hence, cancer vaccine could potentially target any protein that is mutated or expressed differently in cancer cells (27, 28). Since TAAs are also expressed in normal tissue cells, they may be affected by immune tolerance, and therefore immunogenicity may be poor. Moreover, research has demonstrated that tumors generate a distinct antigen known as a tumor-specific antigen (TSAs) as a result of genetic mutations. TSAs are exclusively expressed in particular tumors and are absent on the surface of normal cells (29, 30). TSAs remain unaffected by immune tolerance mechanisms in both central and peripheral systems, enabling them to initiate targeted and potent T cell reactions against cancerous cells (31). Hence, identifying TSAs is crucial for the advancement and utilization of cancer vaccines. However, current studies have found that there are fewer TSAs on the surface of solid tumor cells, which is also a bottleneck limiting tumor immunotherapy.

Whole-cell extracts derived from malignant cells are a key strategy in cancer vaccine development. These extracts, obtained from tumor tissue or cancer cell cultures and rendered non-viable, are recognized by immune cells such as APCs like DCs, macrophages, and natural killer (NK) cells (32). Activated immune cells process the tumor antigens, present them to T cells, and trigger an immune response. CD4+ and CD8+ T cells can recognize and eliminate tumor cells presenting the specific antigen. Whole cell cancer vaccines aim to stimulate a memory response to enhance immune defense and prevent tumor recurrence. DC vaccines, derived from a patient’s peripheral blood and loaded with tumor antigens, are another type of cellular vaccine. These DCs, matured and activated with immune-stimulating agents or TSAs, migrate to lymphatic tissues, where they engage with immune cells, presenting cancer antigens to CD4+ helper T cells and CD8+ cytotoxic T lymphocytes (CTLs), leading to their activation and subsequent elimination of cancer cells (32).

Additionally, recent studies have explored the use of immunohybrids, such as dendritic-tumor cell hybridomas, in prostate cancer immunotherapy. Chowdhury et al. (33) found that the survival of castration-resistant prostate cancer patients treated with these hybridomas was negatively correlated with changes in peripheral blood CD56^bright^CD16− natural killer cells. This highlights the potential of hybrid approaches in enhancing immune responses against prostate cancer. Peptide vaccines and genetically engineered vaccines function similarly in delivering TAAs fragments to B and T lymphocytes through APCs to trigger an adaptive immune response and activated CD8+ T cells start the process of releasing apoptotic factors like perforin, Fas ligand, and granase, leading to tumor cell death (34). Successful therapeutic cancer vaccines rely on providing ample highly immunogenic antigens to the APCs, leading to a robust and lasting CD4+ T helper cell and CD8+ CTLs reaction. Specifically, efforts to enhance APCs activation and maturation focus on improving antigen presentation to generate the best possible T cell reactions. The focus of these methods is on finding, choosing, and confirming new substances that can boost and improve the duration of immune responses from T and B cells that target specific antigens (35).

Despite some advancements in cancer vaccine research, there are still limitations stemming from current research findings. The effectiveness of cancer vaccines is significantly hindered by tumor diversity, immunosuppressive TME, and mechanisms of immune tolerance. Identifying appropriate TAAs and selecting the best adjuvant remain critical for the development and success of cancer vaccines (36). Moreover, the efficacy of this treatment could be constrained by variations in patients’ immune responses and their capacity to identify antigens, necessitating a customized approach for its implementation.

### 3.2 Different types of cancer vaccines are used in the clinical study of PCa treatment

The cancer vaccine plays a crucial role in immunotherapy and holds a distinct position in the treatment of PCa. The FDA gave the green light to sipuleucel-T in April 2010, marking the approval of the initial autologous cell immunotherapy medication utilizing DCs as the primary effector cells. This drug is intended for individuals with asymptomatic or mild mCRPC (37). Sipuleucel-T significantly contributes to the advancement of immunotherapy for PCa. Since then, researchers have developed monocyte vaccines, DCs vaccines, viral vaccines, polypeptide vaccines and DNA/mRNA vaccines for PCa, and achieved initial clinical efficacy (**Table 1**).

**Table 1:** Different vaccines in PCa clinical trials.

Vaccines/types	Clinical Phase	Disease type/Patients	Combination Therapy	Main Findings	Ref.
PROSTVAC/Virus-based vaccines	Phase 3	/104	NA	In most patients, PSA-specific T cells increased significantly after vaccination compared to before vaccination.	J. L. Gulley et al., 2014 (38)
NY-ESO-1/Peptide-based vaccines	Phase 1	mCRPC/14	NA	The treatment of mCRPC with the NY-ESO-1 vaccine is tolerable and appears to slow PSA doubling time.	G. Sonpavde et al., 2014 (39)
PROSTVAC-V -TRICOM/Virus-based vaccines	Phase 2/3	PSA progression without visible metastasis/40	NA	The viral PSA vaccine can be used naïven patients with small disease volumes and can be combined with androgen ablation therapy.	R. S. DiPaola et al., 2015 (40)
PROSTVAC/Virus-based vaccines	Phase 2	mCRPC/8	NA	This study found that PSA-specific CD4+, CD8+ t cells and IgG antibody responses were not detected in mCRPC treated with PROSTVAC.	D. G. McNeel et al., 2015 (41)
20 mixed peptides (KRM-20)/Peptide-based vaccines	Phase 1	CRPC/17	NA	KRM-20 has safety and the ability to enhance CTL activity in the treatment of CRPC.	M. Noguchi et al., 2015 (42)
CD1c/DC-based vaccines	Phase 1	advanced metastatic hormone refractory PCa/12	NA	CD1c-DC-based vaccines are feasible, safe, and well tolerated in the treatment of patients with advanced metastatic PCa.	R. L. Prue et al., 2015 (43)
rPSMA-rSurvivin/DC-based vaccines	Phase 1	HRPC/21	Docetaxel and prednisone	The rPSMA-rSurvivin DC vaccine demonstrated favorable cellular response, disease remission, and no adverse events.	H. B. Xi et al., 2015 (44)
PSA-TRICOM/Virus-based vaccines	Phase 2	mCRPC/44	Sm-153-EDTMP	Combination therapy can significantly reduce PSA in patients.	C. R. Heeryet al. 2016 (45)
n- rmmuc1/DC-based vaccines	Phase 1/2	nmCRPC/17	NA	n-muc1-DC vaccination in nmCRPC patients is safe, induces a significant t cell response, and can significantly improve PSA doubling time.	E. Scheid et al., 2016 (46)
Personalized peptide vaccine (PPV)/Peptide-based vaccines	Phase 2	CRPC/37	dexamethasone	PPV immunotherapy is well tolerated and is associated with prolonged PSA, PFS, and OS in patients with CRPC at the beginning of chemotherapy.	K. Yoshimuraet al. 2016 (47)
Personalized peptide vaccine (PPV)/Peptide-based vaccines	Phase 2	CRPC/70	herbal medicines	PPV combined with herbal medicines have the potential to prevent immunosuppression induced by MDSC or IL-6 during immunotherapy.	N. Koga et al., 2017 (48)
Dcvac/DC-based vaccines	Phase 2	mCRPC/43	docetaxel	mCRPC therapy added to Dcvac was safe and an immune response was detected in approximately half of patients.	P. Kongsted et al., 2017 (49)
hTERT UV1/Peptide-based vaccines	Phase 1/2	mPC/22	GM-CSF	UV1 combined with GM-CSF induced few adverse events and a specific immune response in most patients.	W. Lilleby et al., 2017 (50)
CDCA1/Peptide-based vaccines	Phase 1	CRPC/12	NA	CDCA1 vaccine treatment is tolerable and can effectively induce peptide-specific cytotoxic T lymphocyte responses in CRPC patients.	W. Obara et al., 2017 (51)
BPX101/DC-based vaccines	Phase 1	mCRPC/18	rimiducid (AP1903)	Treatment with BPX101 leads to objective regression of the tumor through immunoupregulation and enhancement of antitumor activity.	G. Sonpavde et al., 2017 (52)
pTVG-HP/DNA vaccines; Sipuleucel-T/DC-based vaccines	–	mCRPC/18	NA	Sipuleucel-T vaccination can enhance and diversify the types of immunity caused by anti-tumor vaccines.	E. Wargowski et al., 2018 (53)
MVI-816/DNA vaccines	Phase 2	CSPC/99	GM-CSF	pTVG-HP treatment did not show any improvement in 2-year metastasis-free survival in castration-sensitive PCa patients.	D. G. McNeel et al., 2019 (54)
PROSTVAC/Virus-based vaccines	Phase 2	Localized PCa/27	NA	PROSTVAC can induce not only tumor immune response, but also peripheral immune response.	H. Abdul Sater et al., 2020 (55)
pTVG-AR, MVI-118/DNA vaccines	Phase 1	mCSPC/40	NA	pTVG-AR is safe and immunoactive in patients with mCSPC and may delay the time of castration resistance.	C. E. Kyriakopoulos et al., 2020 (56)
KRM-20/Peptide-based vaccines	Phase 2	CRPC/51	docetaxel	In CRPC patients with lymphocytes ≥26% or PSA levels < 11.2 ng/ml, KRM-20 combined with docetaxel is beneficial.	M. Noguchiet al. 2020 (57)
RhoC/Peptide-based vaccines	Phase 1/2	Patients with radical prostatectomy/22	NA	RhoC vaccine can induce effective and durable T cell immunity, which may delay or prevent tumor recurrence and metastasis formation.	J. Schuhmacheret al. 2020 (58)
Ad5 PSA/MUC-1/brachyury vaccine/mRNA vaccines	Phase 2	mCRPC/	NA	The Ad5 PSA/MAC-1/brachyury vaccine was well tolerated and met the primary endpoint without dose-limiting toxicity.	M. Bilusic et al., 2021 (59)
Sipuleucel-T/DC-based vaccines	Phase 1	mCRPC/37	atezolizumab	sipuleucel-T in combination with atezolizumab was well tolerated and its safety was comparable to that of each drug administered alone.	T. Dorff et al., 2021 (60)
GX301/Peptide-based vaccines	Phase 2	mCRPC/98	NA	The GX301 cancer vaccine is safe and immunogenic in patients with mCRPC.	G. Filaci et al., 2021 (61)
human leukocyte antigen (HLA)-A24/Peptide-based vaccines	Phase 3	CRPC/306	NA	PPV did not prolong OS in patients with CRPC, however, patients with a lower proportion of neutrophils or a higher proportion of lymphocytes may benefit from PPV treatment for survival.	M. Noguchi et al., 2021 (62)
Sipuleucel-T/DC-based vaccines	Phase 2	mCRPC/50	ipilimumab	Sipuleucel-T vaccine combined with ipilimumab has low clinical activity. Blocking CTLA-4 after sipuleucel-T treatment did not change the antigen-specific response.	M. Sinha et al., 2021 (63)
MVI-816/DNA vaccines	Phase 2	mCRPC/	Pembrolizumab	MVI-816 combined with ICIs can increase tumor-specific T cells and favorable 6-month disease control rate.	D. G. McNeel et al., 2022 (64)
DC-based vaccines	Phase 1/2	High-risk PCa/22	Robot-assisted laparoscopic prostatectomy (RALP)	DC vaccine response was associated with a reduced incidence of BCR.	A. M. A. Tryggestad et al., 2022 (65)
PrCa vir/DNA vaccines	Phase 1	mCRPC and BCR/91	NA	PrCa vir showed a similar safety profile to ICIs therapy and produced modest antitumor activity in BCR patients who did not use ADT.	K. A. Autioet al. 2023 (66)
hTERT/Peptide-based vaccines	Phase 2	primary metastatic hormone-sensitive PCa/22	ADT and radiotherapy.	hTERT vaccination may be clinically beneficial in a subgroup of patients with primary metastatic hormone-sensitive PCa treated with ADT and radiotherapy.	W. Lilleby et al., 2023 (67)
PROSTVAC/Virus-based vaccines	Phase 2	NmCRPC/64	flutamide	Compared with flutamide alone, the combination of flutamide and PROSTVAC did not improve the outcome of patients with nmCRPC.	R. A. Madan et al., 2023 (68)
pTVG-HP/DNA vaccines	Phase 2	mCRPCs/19	Nivolumab	pTVG-HP vaccination combined with Nivolumab was safe and immunologically active, extending the time of disease progression but not eradicating the disease.	D. G. McNeel et al., 2023 (69)
PROSTVAC/Virus-based vaccines	Phase 2	localized PCa/154	NA	The vaccinated participants did not show more favorable outcomes than the control group.	J. K. Parsons et al., 2023 (70)
hTERT/Peptide-based vaccines	Phase 1	advanced solid tumors (Colorectal cancer and PCa)/29	targeting Tregs and cyclooxygenase-2 (COX2)-mediated immunosuppression	Median PFS was 9 months, and 24% of patients had PFS ≥6 months.	N. Zareian et al., 2024 (71)

#### 3.2.1 DCs vaccines

In recent years, researchers have endeavored to utilize cancer vaccine strategies for the treatment of mCRPC, resulting in promising initial efficacy. The FDA approved Sipuleucel-T (Provenge) in April 2010 as the initial tumor immunotherapy medication, offering a ray of hope for individuals with mCRPC (72). Sipuleucel-T is an autologous cell immunotherapy based on DCs. A multicenter Phase 3 clinical trial has shown that Sipuleucel-T can significantly prolong survival in patients with mCRPC (73). The study included 512 patients, 341 of whom received Sipuleucel-T for 1 month and 171 of whom received a placebo control. The study revealed that individuals receiving Sipuleucel-T treatment experienced a 22% decrease in mortality risk and a median survival increase of 4.1 months. Following this study, Sipuleucel-T was granted FDA authorization for managing asymptomatic or mild mCRPC, marking the beginning of a fresh period of immunotherapy for PCa.

The safety and efficacy of other types of DCs vaccines against mCRPC have also been preliminatively explored. R. L. Prue et al. (43) conducted a phase I clinical study to evaluate the safety of blood-derived DCs (BDC) purified with tumor-specific peptide CD1c in patients with mCRPC. Finally, the CD1c BDC vaccine was administered to 12 mCRPC patients. The vaccine was well tolerated in all patients and no serious adverse events were detected. Similarly, BPX101 is a second-generation antigen-targeted autologous DCs vaccine, and its safety and activity were also reported in a Phase I trial (52). Eighteen patients with mCRPC were enrolled and given three doses of BPX101 subcutaneously. The results of this study found no dose-limiting toxicity of BPX101. Immunoupregulation and significant antitumor activity were observed in patients with decreased prostate-specific antigen (PSA), while objective tumor regression was also observed. Moreover, H. B. Xi et al. (44) applied DCs vaccine loaded with recombinant prostata-specific membrane antigen (rPSMA) and recombinant surviving (rSurvivin) peptide to 21 patients with hormone-refractory prostate cancer (HRPC). Patients were randomized into two groups, with docetaxel treatment as a control. The study also found that the DCs vaccine was well tolerated. The DCs vaccine induced delayed immune responses in all patients. Compared with the control group, the response rate was 72.7% vs 45.4%, and the immune-related response was 54.5% vs 27.2%. Therefore, the above studies have shown that DCs vaccine is not only well tolerated, but also has a good immune response.

Additionally, researchers are endeavoring to employ the DCs vaccine in patients with localized PCa. MUC1 is a surface glycoprotein expressed in ductal epithelial cells. Tumors undergo malignant transformation resulting in increased levels of MUC1, categorized as T or Tn tumor antigens based on the type of carbohydrate present. Tn-MUC1, containing Tn glycans, is a promising candidate for immunotherapy (74). E. Scheid et al. (46) applied autologous DCs loaded with Tn-MUC1 to non-mCRPC and discussed its safety and efficacy. The Tn-MUC1-DCs vaccine was tested on 17 patients with nmCRPC in a Phase I/II clinical trial. There was a significant improvement in PSA doubling time in 11 out of 16 patients who were evaluated (*P* = 0.037). Five of the seven patients had a significant immune response. Tn-MUC1-DCs vaccine in patients with nmCRPC appears to be safe and able to induce a significant T cell response. In addition, one researcher also tried to apply this technology to the adjuvant treatment of PCa patients. Furthermore, A. M. A. Tryggestad et al. (65) discussed the value of personalized DCs vaccine in reducing biochemical recurrence (BCR) in patients after robot-assisted laparoscopic prostatectomy. Twenty individuals diagnosed with high-risk PCa and undetectable prostate-specific antigen levels were administered the DCs vaccine for a period of three years or until experiencing biochemical recurrence. Out of the 20 individuals, 11 were determined to have no evidence of BCR after a median follow-up of 96 months (ranging from 84 to 99 months). All patients who developed BCR remained stable for a median of 99 months. The study is the first to use DCs vaccine as an adjunct postoperative treatment for high-risk PCa, and the results have been encouraging. Therefore, attempts should be made to apply the DCs vaccine in a larger cohort of high-risk PCa patients to further investigate its value for BCR treatment.

In order to better exert the efficacy of DCs vaccine in the treatment of PCa, some researchers have also tried to use a combination of other tumor treatment strategies. P. Kongsted et al. (49) investigated whether adding DCs vaccine to docetaxel treated mCRPC patients induced an immune response. A total of 43 patients were randomly allocated to either receive docetaxel by itself or in combination with DCs vaccine. PSA, progression-free survival (PFS), and disease-specific survival (DSS) responses did not show any notable variations. TAAs-specific or vaccine-specific immune responses were 50% and 78% in the combination treatment group respectively (75, 76). Therefore, more clinical studies are needed in the future to explore the clinical efficacy of docetaxel combined with DCs vaccine. Moreover, the researchers propose that the concurrent administration of ICIs and cancer vaccines has the potential to modulate the immune system, leading to sustained activation of T cells and a durable immunotherapeutic effect. T. Dorff et al. (60) enrolled 37 patients with asymptomatic or mild symptoms of mCRPC and conducted an initial clinical trial (NCT03024216). Random assignment determined which enrolled patients would receive sequential treatment with either atezolizumab + sipuleucel-T or sipuleucel-T + atezolizumab. The safety and tolerability of atezolizumab when combined with sipuleucel-T in various sequences was confirmed, with immunosurveillance studies indicating the potential benefits of this combination. Similarly, M. Sinha et al. (63) investigated whether administration of ipilimumab following sipuleucel-T treatment could alter immune and clinical response to this treatment. A total of 50 mCRPC patients enrolled in a clinical trial (NCT01804465) who were treated with ipilimumab either immediately after completing sipleul-T or with a delay of 3 weeks. The integrated therapy approach was determined to be well received with no unforeseen negative effects. Six patients exhibited positive clinical responses, with three of them maintaining the response for over 3 months. The use of multiple treatments did lead to the activation of CD4 and CD8 T cells, especially in the early treatment schedule. The above studies have conducted a preliminary exploration of DCs vaccine combined with other therapeutic measures. Further exploration of the cancer vaccine’s mechanism of action in the TME is necessary for widespread translation into clinical practice.

#### 3.2.2 Peptide-based vaccines

##### 3.2.2.1 Simple peptide targeting

Cancer vaccines based on peptides can be used in cancer treatment, especially against tumor growth and the tumor recurrence (77). Over the past decade, peptide cancer vaccines have been evaluated in multiple tumor models as a component of research in cancer immunotherapy (78). Cancer vaccines utilize two primary categories of peptides: TAAs and TSAs, which are capable of being processed by APCs and displayed to immune cells. The activation and proliferation of CD8+ cytotoxic T cells occur when they identify the presented peptides on MHC Class I molecules, leading to the destruction of tumor cells. CD4+ helper T cells identify peptides on MHC Class II molecules and transmit signals to other immune cells to aid in inhibiting tumor growth. B cells are activated by the peptides presented, resulting in the production of specific antibodies against TAAs, which ultimately induce a memory response to enhance immune protection (79).

NY-ESO-1, a tumor antigen found in testicular cancer, is present in both tumors and normal testis, but is not found in other normal tissues. NY-ESO-1 is the most potent antigen among the testicular antigens identified so far, stimulating both antibody production and robust cellular immune reactions in patients (80, 81). G. Sonpavde et al. (39) conducted a Phase I clinical trial to assess the efficacy of the NY-ESO-1 peptide vaccine in mCRPC. The study comprised nine patients, four of whom had received prior treatment with docetaxel. A higher frequency of specific T cell response was found in docetaxel pretreated patients in the NY-ESO-1 treatment group (4/4 vs 2/5). The research demonstrates that mCRPC patients can tolerate the NY-ESO-1 peptide, and it elicits antigen-specific T cell responses more frequently in patients undergoing chemotherapy. CDCA1 is upregulated as an oncogene in PCa. W. Obara et al. (51) assessed the effectiveness of a CDCA1 peptide vaccine for treating CRPC in a phase I (NCT01225471). This study included 12 patients with CRPC who had failed docetaxel chemotherapy. Patients received subcutaneous injections of CDCA1 peptide in a dose-increasing manner. The CDCA1 peptide vaccine was determined to be well received with no significant negative effects observed. Patients who had a longer survival time showed a specific CTL response to CDCA1 peptide, with a median Overall survival (OS) of 11.0 months. This study demonstrated that CDCA1-derived peptide vaccines can effectively induce peptide-specific CTL responses in patients with CRPC. RhoC, a member of the Ras homologous gene family C (RhoC), demonstrates increased expression levels in advanced solid tumors, metastatic tumors, and cancer stem cells. J. Schuhmacher et al. (58) examined the safety, tolerability, and impact of RhoC peptide vaccine on the immune system in individuals with PCa (NCT03199872). The study included 22 individuals who had previously received radical prostatectomy, with a treatment period lasting 30 weeks. Most patients (18 out of 21) exhibited a robust CD4 T cell reaction to the vaccine, which persisted for a minimum of 10 months following the final dose, as per the findings. Furthermore, the vaccine was well received with no severe treatment-related adverse events reported. According to this research, immunization against RhoC peptide vaccine could potentially postpone or hinder the reappearance and spread of tumors. Homogeneously, G. Filaci et al. (61) developed GX301, a cancer vaccine that targets telomerase. The safety and immune response of the GX301 vaccine were evaluated in patients with metastatic castration-resistant PCa (NCT02293707). Ninety-eight patients were randomly vaccinated. 54% of patients exhibited an immune response overall, with 95% demonstrating at least one immune response specific to the vaccine. The data suggest that the GX301 cancer vaccine is well-tolerated and induces an immune response in individuals with advanced castration-resistant PCa. These studies indicate that the use of fetal fragments of tumor antigens as cancer vaccines has good safety and specific immune response in the treatment of PCa. Further clinical studies are expected to investigate its value in improving the clinical prognosis of patients.

In recent years, researchers have developed a human telomerase reverse transcriptase(hTERT) cancer vaccine, which is widely used in the immunotherapy of tumors (50, 82, 83). W. Lilleby et al. (50) assessed the safety and immune reaction of the new hTERT peptide vaccine (UV1) in patients with recently diagnosed metastatic hormone-naïve PCa. Twenty-two recently diagnosed patients were included in the study, receiving three different doses of UV1 administered intradermally along with granulocyte macrophage stimulating factor (GM-CSF). By the conclusion of the nine-month observation period, a total of 17 patients showed signs of clinical stability. UV1 and GM-CSF therapy had minimal side effects and triggered a targeted immune reaction in most patients who were not HLA type-selected. The moderate amount of UV1 led to the most significant and quickest UV1-targeted immune reaction while maintaining a safe profile. More recently, W. Lilleby et al. (67) evaluated the long-term immune system response and effectiveness of patients in this study. Nine out of the 22 patients were alive at the most recent check-in. There was no progression in 6 cases, castration-resistant disease in 2 cases and castration-refractory disease in 1 case. PSA progression had a median time of 21 months, while OS and PCa-specific survival had median times of 62 and 84 months, respectively. Absence of immune reaction is a separate factor that increases the risk of death from PCa. The connection implies a potential medical advantage of hTERT immunization in a specific group of individuals with initial metastatic hormone-responsive PCa who have undergone androgen deprivation therapy and radiotherapy. Furthermore, N. Zareian et al. (71) utilized hTERT peptide in conjunction with treatment aimed at inhibiting Tregs and COX2-induced immunosuppression. In the Phase 1 study, a 7-peptide library derived from hTERT was utilized, along with oral low-dose cyclophosphamide for Tregs regulation and the COX2 inhibitor celecoxib. 29 patients with advanced PCa patients were included in the study. Median PFS was 9 weeks, and 24% of patients had PFS≥6 months. The immunophenotype indicated an increase in CD4(+) and CD8(+) T cells following inoculation, displaying characteristics of effector cells. Diminished numbers of PD-1 positive CTLs are increasing in individuals who have received vaccinations. This vaccine combination regimen is safe and associated with an antigen-specific immune response. Due to its robust and enduring capacity to stimulate tumor immune response, the hTERT peptide vaccine shows promising potential for the treatment of PCa in clinical settings.

##### 3.2.2.2 Mixed peptide targeting

The variability within the human T cell population and the varied expression of TAAs suggest that effective cancer vaccines should elicit a broad spectrum of cytotoxic T lymphocyte responses, a strategy that can be achieved by targeting multiple TAAs with vaccination. M. Noguchi et al. (42) examined the safety and immune responses of a cancer vaccine containing 20 assorted peptides (KRM-20) aimed at stimulating CTL against 12 various TAAs in individuals with CRPC. Seventeen patients were given three different doses of KRM-20 that were randomly assigned. The findings indicated that there were no significant adverse effects from the medication. Clinical reactions to levels of prostate-specific antigen showed partial response in 2 instances, stability in 5 instances, and worsening condition in 10 instances. This study confirmed the safety of KRM-20 in PCa treatment and its ability to enhance CTL activity, however, its efficacy was not ideal, the authors attributed to the dose of treatment. The absence of further research on the dosage could also stem from the lack of precision or weak immune response of the target associated with KRM-20. Since then, the efficacy of KRM-20 in combination with docetaxel has been investigated. M. Noguchi et al. (57) explored if the addition of KRM-20 to docetaxel could improve the effectiveness against CRPC. Random assignment was used to allocate eligible patients to two groups: one receiving KRM-20 with docetaxel (n=25) and the other receiving a placebo with docetaxel (n=26). This study demonstrated that the addition of KRM-20 to CRPC therapy is safe, and the decrease in PSA and the increase in HLA-matched peptide-specific CTL and IgG responses suggest the potential of KRM-20 in the treatment of CRPC. These studies indicate that the mixed peptide vaccine has a good safety profile in the treatment of PCa, but does not show an advantage in improving patient outcomes. Due to the characteristics of targeting multiple antigens, this vaccine can solve the problem of differential expression of target antigens between individuals, and has great economic value. Therefore, more research is needed to explore the mechanism of action of this vaccine in PCa patients.

##### 3.2.2.3 Individualized peptide

Due to the variability in the expression of target antigens among individuals, the implementation of immunotherapy necessitates the screening of appropriate candidates. Consequently, each immunotherapy approach is tailored to the individual, with ongoing efforts by researchers to develop personalized cancer vaccines specifically for the treatment of PCa. K. Yoshimura et al. (47) evaluated the safety and clinical efficacy of immunotherapy with personalized peptide vaccine (PPV). A phase 2 randomized controlled trial was conducted to assess the efficacy of combining PPV immunotherapy with dexamethasone in the treatment of chemotherapy-naive patients with CRPC. Four HLA-matched peptides were chosen according to the reaction of existing immunoglobulin G to 24 stored peptides and administered biweekly. Peptide vaccine was administered to thirty-seven patients, while thirty-five patients were treated with dexamethasone alone. PFS in inoculation group was significantly longer than dexamethasone group (22.0 months vs 7.0 months; *P* = 0.0076). The vaccinated group had a notably longer median survival time compared to the non-vaccinated group (73.9 months vs 34.9 months; *P* = 0.00084). PPV immunotherapy is well tolerated and is associated with prolonged PFS and OS in patients with CRPC at the beginning of chemotherapy. Moreover, M. Noguchi et al. (62) conducted a Phase III randomized trial of a PPV in HLA-A24 positive CRPC patients who had failed docetaxel chemotherapy. Participants were assigned randomly to either receive PPV or placebo at a ratio of 2:1. Four out of the 12 peptides stored were chosen based on existing levels of peptide-specific immunoglobulin G or a placebo and were administered subcutaneously six times per week until the disease advanced. A total of 306 patients were included in the final analysis. Analysis of intergroup effectiveness indicated that PPV did not extend overall survival in individuals with CRPC who experienced disease progression following docetaxel chemotherapy and tested positive for HLA-A24. Analysis of subgroups indicated that individuals with a low neutrophil ratio or high lymphocyte ratio at the beginning of the study may experience improved survival outcomes with PPV therapy. The findings indicate that personalized peptide vaccines may necessitate the identification of suitable candidates. Additionally, characterizing the tumor immune microenvironment at various stages of tumor progression could aid in the identification of suitable candidates for personalized peptide vaccines.

Furthermore, initial efforts have been made to explore the efficacy of combining herbal medicines (HM) with personalized vaccines in the treatment of PCa. N. Koga et al. (48) explored the immunological efficacy of HM combined with personalized peptide vaccine (PPV) in the treatment of CRPC through a phase II clinical study. Seventy patients with CRPC were divided into PPV + HM and PPV alone groups. Based on the human leukocyte antigen type and the level of antigen specific IgG titer in patients prior to receiving PPV treatment, 2-4 peptides were chosen from a pool of 31 peptides derived from cancer antigens, followed by 8 injections of PPV. The results of this study showed that monocyte medullary derived suppressor cell (Mo-MDSC) frequency and serum IL-6 level were stable during PPV combined with HM treatment. In contrast, Mo-MDSC frequency and IL-6 levels were significantly increased in the PPV group alone. The findings indicate that the mixture of HM could have the ability to inhibit immunosuppression caused by Mo-MDSC or IL-6 in the course of immunotherapy. Further investigation is required to validate the findings of this research. The study indicates that the integration of personalized vaccines with immune modulators, such as HM, could potentially serve as a novel approach for enhancing PCa immunotherapy.

#### 3.2.3 Nucleic acid-based vaccines

DNA and RNA cancer vaccines work by delivering DNA or RNA molecules that contain instructions for TAAs. Cells in the body absorb the introduced DNA or RNA, leading to the production of TAAs. APCs cells take up TAAs and use MHC molecules to present it on their surface. CD8+ CTLs identify TAAs on MHC class I molecules, resulting in their stimulation and proliferation. CD4+ T helper cells identify TAAs on MHC Class II molecules and transmit activation signals to other cells of the immune system. B cells can be activated by TAAs presented by DCs, resulting in the production of antibodies against TAAs. Antibodies can bind directly to TAAs of tumor cells, promote their destruction, and induce a memory response to enhance immune protection.

Prostate acid phosphatase (PAP) serves as a prostate tumor antigen and is the specific target of sipuleucel-T, the sole anti-cancer vaccine approved by the FDA. E. Wargowski et al. (53) sought to assess whether a DNA vaccine encoding PAP (pTVG-HP) could enhance specific immunity in patients with mCRPC. Eighteen patients were randomly assigned to two groups: one group received monocyte T therapy alone, while the other group received intradermal pTVG-HP DNA vaccine afterwards. The treatment protocol was completed by 11 out of 18 patients. No adverse events above Grade 2 were detected in relation to treatment. Specific T cell responses to PAP with a Th1 bias were found in 11 of the 18 individuals, and there was no significant difference between the groups in the study. A high titer antibody response to PAP was detected in patients receiving pTVG-HP booster immunization. These findings suggest that from the perspective of T cell and humoral immunity, DNA as a pre-booster vaccination can enhance and diversify the types of immunity induced by anti-cancer vaccines. However, previous study has shown that pTVG-HP does not improve 2-year metastasis-free survival (MFS) in castration-sensitive PCa (54). Subsequently, D. G. McNeel et al. (64) found that the combination of ICIs with pTVG-HP increased tumor-specific T cells in patients with mCRPC and resulted in a favorable 6-month disease control rate. Recently, D. G. McNeel et al. (69) reported the efficacy of DNA vaccine pTVG-HP combined with pembrolizumab in mCRPC (NCT03600350). Of the 19 patients enrolled, PSA decreased by >50% in 4/19(21%) patients. In this population, pembrolizumab combined with pTVG-HP vaccination was safe and immunologically active, extending the time of disease progression but not eradicating the disease.

Studies conducted before clinical trials have indicated that a DNA vaccine (pTVG-AR) containing the ligand-binding domain of the androgen receptor (AR LBD) boosts CD8+ cells activization that target specific antigens, slows down the advancement of PCa and the development of castration-resistant disease, and extends the lifespan of mice with tumors. A multicenter Phase I trial assessed the effectiveness of the vaccine (56). Patients diagnosed with metastatic castration-sensitive prostate cancer (mCSPC) received treatment with pTVG-AR; 27 individuals successfully finished the treatment regimen, and 11 patients (28%) experienced a PSA progression event prior to reaching 18 months. Individuals possessing T cell immunity exhibited notably extended PFS compared to those lacking immunity (HR = 0.01; 95% CI, 0.0-0.21; *P* = 0.003). The research showed that pTVG-AR is both well-tolerated and stimulates the immune system in individuals with mCSPC. The correlation of immune response with PFS indicates that therapies could potentially extend the time before developing resistance to castration. The research shows how pTVG-AR could enhance results for individuals with mCSPC, but additional prospective randomized controlled trials are necessary for validation.

#### 3.2.4 Virus-based vaccines

A viral vaccine is a viral vector that delivers recombinant genes targeting TAAs. These viral vectors expressing recombinant genes play a role by infecting epithelial cells. After cell death, the cell fragments containing target antigens are recognized and processed by APCs and presented to CD4+ and CD8+ T cells to induce immune response, and then the activated immune system can kill tumors.

PROSTVAC is a therapeutic vaccine based on two PSA-encoding recombinant poxvirus vectors, in which vaccinia virus vectors serve as initial immunization, followed by avian pox virus booster immunization, and the two recombinant viruses simultaneously encode three co-stimulatory molecules (B71, ICAM-1, and AF-3), also known as TRICOM. PROSTVAC can trigger an immune response against tumors and in the surrounding area, leading to immune cells entering the tumor environment and destroying cancer cells (55). J. L. Gulley et al. (38) investigated the efficacy of the PROSTVAC vaccine for PCa through the implementation of a Phase III clinical trial. Among the cohort of 104 individuals studied for T cell response, 57% (59/104) demonstrated a greater than twofold increase in PSA-specific T cells one month after vaccination compared to pre-vaccination levels. Additionally, 68% (19/28) of individuals exhibited an immune response to tumor-related antigens not present in the vaccine post-vaccination. These findings suggest that PSA antibodies do not influence changes in PSA levels following vaccination. Assessing the systemic immune reaction to PSA might not accurately reflect the actual therapeutic immune response. However, studies by other investigators have not found a tumor-specific immune response induced by the PROSTVAC vaccine in PCa treatment. J. K. Parsons et al. (70) explored clinical indicators for active monitoring of immune response to PROSTVAC vaccine and disease progression in patients with localized PCa. The research involved 154 males diagnosed with low to moderate-risk PCa. Included patients were randomly divided (2:1) into treatment and control groups, receiving PROSTVAC vaccine and empty fowlpox vector (EV) respectively. The study results showed that PROSTVAC did not elicit a better response in prostate tissue or peripheral T cells compared to EV. Similarly, D. G. McNeel et al. (41) assessed the effectiveness of the vaccine in CRPC patients undergoing docetaxel chemotherapy. Eight patients with mCRPC received treatment, but no immune responses targeting PSA-specific CD4+ and CD8+ T cells or PSA-specific IgG antibodies were observed. Further research is required to validate the effectiveness of the PROSTVAC vaccine for treating PCa.

Multi-target viral vaccines for PCa treatment have also been developed and used in clinical studies. Researchers have also developed a multi-target viral vaccine for PCa treatment. M. Bilusic et al. (59) developed an innovative viral vaccine utilizing adenovirus 5 (Ad5) carriers to target three TAAs: PSA, brachyury, and MUC-1. These transgenes have epitope alterations that enhance the activation of CD8+ T cells. Safety and efficacy were determined through a Phase 1 clinical study (NCT03481816). A total of eighteen individuals diagnosed with mCRPC participated in the study and were administered at least one dose of the vaccine. The participants responded positively to the Ad5 PSA/MUC-1/brachyury vaccine. Primary endpoint was met without dose-limiting toxicity. The vaccine showed clinical activity, including partial responses and PSA responses in five patients. Three patients with prolonged PSA response received palliative radiotherapy. Additional research is required to assess the therapeutic advantages and immune response of this vaccine when used alongside other immuno-oncology medications.

In addition, PROSTVAC is being tested in combination with other strategies for PCa. The E9802 trial is a Phase 2 study involving multiple institutions, aiming to assess the safety and effectiveness of PROSTVAC in combination with ADT for patients without significant metastasis but experiencing PSA progression (40). Of the 27 patients, 20 achieved complete response at 7 months. This study confirms that the viral PSA vaccine can be used in patients with smaller disease volumes and that combined with ADT may result in better disease response rates. This information provides evidence for the potential benefits of implementing vaccine treatment at an early stage of PCa in future research, aiming to lessen the impact of the disease. Samarium-153-ethyleneenetetramethylene phosphonate (Sm-153-EDTMP) is a radiopharmaceutical that binds to osteoblast lesions and releases beta particles that can cause local tumor cell destruction. C. R. Heery et al. (45) tried Sm-153-EDTMP combined with PROSTVAC for the treatment of mCRPC. In this multicenter trial’s 2 phase, patients were randomly assigned to receive either Sm-153-EDTMP by itself or in conjunction with the PROSTVAC vaccine. The study findings indicated that the median PFS for Sm-153-EDTMP was 1.7 months for the Sm-153-EDTMP group and 3.7 months for the combination group, with a statistically significant difference (*P* = 0.041). None of the patients in the Sm-153-EDTMP group experienced PSA reductions greater than 30%, whereas three out of four patients in the combined group had PSA reductions exceeding 50%. The findings offer a theoretical foundation for utilizing novel radiopharmaceuticals in conjunction with PROSTVAC for treating PCa. However, a recent study confirmed that androgen receptor antagonist (ARAs) flurtamide combined with PROSTVAC does not improve outcomes in patients with nmCRPC (68). Therefore, more RCT studies are needed in the future to confirm the value of the viral vaccine combined with anti-male treatment strategy in the treatment of PCa.

PF-06753512(PrCa VBIR) is a hybrid viral vaccine based for the treatment of PCa. The PrCa VBIR combines the functions of a vaccine and an immune checkpoint inhibitor, consisting primarily of an adenovirus vector that expresses PSA, PSMA, prostatic stem cell antigen (PSCA), and tremelimumab. K. A. Autio et al. (66) investigated the efficacy of PF-06753512 in mCRPC through Phase I clinical trial. The study involved 91 participants, with PSMA eliciting a response from 88.0% of antigen-specific T lymphocytes, PSA from 84.0%, and PSCA from 80.0%. PrCa VBIR generally showed similar safety signals as other ICIs combination tests. Significant side effects were found in some patients with biochemical relapse. It triggered immune response against specific antigens in all groups and showed some effectiveness in fighting tumors in patients experiencing biochemical relapse without ADT. This study suggests that hybrid virus vaccines have potential in the treatment of PCa.

## 4 Immune checkpoint inhibitors are used in the treatment of PCa

### 4.1 ICIs

**Figure 3:**
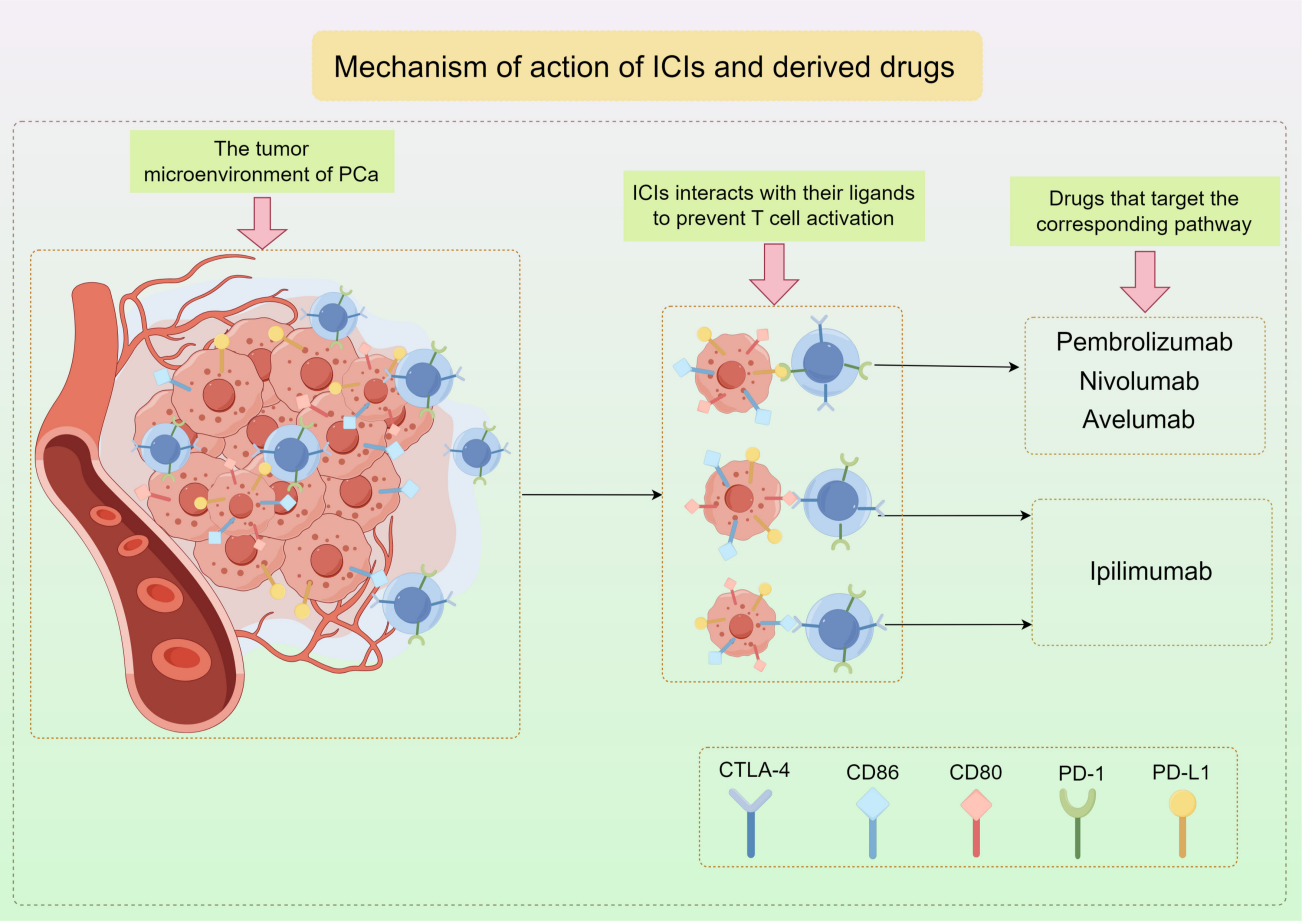
The mechanism of action of ICIs and the representative drugs used in PCa by Figdraw.

PD-1 is found on activated T cells and NK cells, and when it binds to PD-L1, it can block T cell activation, converting naive T cells into Tregs, which helps prevent the immune system from attacking normal cells in a specific immune response (84–86). Tumor cells that have PD-L1 utilize this pathway in order to evade the immune response against tumors by T cells. Cytotoxic T-lymphocyte-associated protein 4 (CTLA-4), a protein found on T cells similar to PD-1, interacts with CD80 and CD86 ligands to prevent T cell activation (**Figure 3**) (87). Consequently, therapies targeting these pathways have become more prevalent in contemporary cancer treatment. Immunotherapy targeting checkpoints has greatly enhanced the management of numerous solid tumors, notably lung cancer, melanoma, and renal cell carcinoma (88–93).

### 4.2 Clinical study of ICIs in PCa

The utilization of ICIs in PCa presents a significant challenge due to the inherent weakness of the immune response in PCa and the low TMB (94). Tumors characterized by a high mutation burden are more likely to exhibit a favorable response to ICIs due to the presence of neoantigens that can be recognized by reactivated T cells, whereas tumors with a low mutation burden typically demonstrate restricted clinical efficacy. This means that fewer immune cells, including T cells, enter the tumor tissue. In addition, the hypoxic region of the TME in PCa leads to poor infiltration ability of T cells into tumor tissue. These hypoxic regions inhibit T cell function through multiple mechanisms, including acidic pH, nutrient depletion, increased adenosine expression, and PD-L1 (95, 96), and also promotes phenotypic transformation of immature myeloid cells into myeloid suppressor cells (MDSCs) and tumor-related macrophages, which made the immunosuppressive characteristics of the TME more obvious (95). However, recent studies have suggested that therapy directed at PD-L1 may hold potential for the treatment of PCa. The expression of PD-L1 in PCa tissues plays a crucial role in determining the suitability of immune checkpoint inhibitors for PCa therapy. Several preclinical research studies have examined the expression of PD-L1 in PCa samples to assess the effectiveness of checkpoint inhibitor treatment in individuals with PCa. A study utilizing immunohistochemistry (IHC) to evaluate tumor scores on 402 prostatectomy samples found that 92% (371/402) exhibited positive PD-L1 staining in tumor epithelial cells, while 59% (236/402) had high intensity PD-L1 scores. While there was a strong correlation between high-density PD-1 + lymphocytes and reduced clinical survival, this research did not discover a significant link between PD-L1 expression and the prognosis of PCa (97). A separate research study utilizing primary PCa samples from two distinct cohorts revealed that, 50 to 60 percent of cases exhibited moderate to high levels of PD-L1 expression in immunohistochemistry staining. The presence of PD-L1 positivity was determined to be a prognostic indicator for biochemical recurrence through multivariate Cox analysis (*P*= 0.007) (98). In conclusion, ICIs pose distinct challenges and hold considerable potential in the management of PCa. Recent research endeavors have focused on the application of ICIs targeting PD-1/PD-L1 and CTLA-4 in the treatment of PCa, demonstrating encouraging preliminary outcomes (**Table 2**).

**Table 2:** Different ICIs in PCa clinical trials.

Drugs	Clinical Phase	Disease type/Patients	Combination Therapy	Main Findings	Ref.
Ipilimumab	Phase 1	mCRPC/30	PROSTVAC vaccines	An increase in natural killer cells was associated with longer survival after vaccination compared to before vaccination.	C. Jochems et al., 2014 (99)
Ipilimumab	Phase 3	mCRPC/799	bone-directed radiotherapy	Ipilimumab has not been shown to improve OS, but there are indications that it has some antitumor activity.	E. D. Kwon et al., 2014 (100)
Ipilimumab	Phase 3	mCRPC/400	NA	Ipilimumab does not improve OS in patients with metastatic castration-resistant PCa, however, increases in PFS and prostate-specific antigen response rate suggest antitumor activity.	T. M. Beeret al. 2017 (101)
Ipilimumab	Phase 3	mCRPC/799	radiotherapy	Ipilimumab combined with radiotherapy can significantly improve patients’ OS.	K. Fizazi et al., 2020 (102)
Nivolumab/Ipilimumab	–	mCRPC/	NA	The combination treatment strategy showed an objective response rate of 10% to 25%, and treatment-related adverse events remained high.	P. Sharmaet al. 2020 (103)
Nivolumab plus Ipilimumab	Phase 2	mCRPC/30	NA	Nivolumab in combination with ipilimumab has shown only modest activity in PCa patients with ar-v7 expression.	E. Shenderov et al., 2021 (104)
Sipuleucel-T/DC-based vaccines	Phase 2	mCRPC/50	ipilimumab	Sipuleucel-T vaccine combined with ipilimumab has low clinical activity. Blocking CTLA-4 after sipuleucel-T treatment did not change the antigen-specific response.	M. Sinha et al., 2021 (63)
Avelumab	Phase 1	mCRPC/26	carboplatin	Avelumab in combination with carboplatin has an acceptable safety profile and is associated with prolonged OS.	A. Rodriguez-Vida et al., 2023 (105)
Pembrolizumab	Phase 2	mCRPC/10	enzalutamide	Pembrolizumab showed favorable antitumor activity in some patients (3 out of 10).	J. N. Graff et al., 2016 (106)
Pembrolizumab	Phase 2	mCRPC/258	NA	Pembrolizumab therapy showed antitumor activity and an acceptable safety profile in mCRPC patients previously treated with docetaxel.	E. S. Antonarakis et al., 2020 (107)
Pembrolizumab	Phase 2	mCRPC/28	enzalutamide	Pembrolizumab combined with enzalutamide has good antitumor activity in mCRPC.	J. N. Graffet al. 2020 (108)
Pembrolizumab	Phase 1	oligometastatic hormone-sensitive PCa/12	cryotherapy	Prostate cryoablation combined with pembrolizumab is well tolerated and has no safety concerns in patients with low-metastatic PCa.	A. E. Ross et al., 2020 (109)
Pembrolizumab	Phase 2	mCRPC/60	MVI-816/DNA vaccines	MVI-816 combined with ICIs can increase tumor-specific T cells and favorable 6-month disease control rate.	D. G. McNeel et al., 2022 (64)
Pembrolizumab	Phase 1/2	mCRPC/104	docetaxel and prednisone	Pembrolizumab in combination with docetaxel and prednisone has shown antitumor activity and a manageable safety profile in patients with mCRPC.	E. Y. Yu et al., 2022 (110)
Pembrolizumab	Phase 2	mCRPC/43	(177) Lu-PSMA-617	A single dose of (177) Lu-PSMA-617 following pembrolizumab maintenance therapy was safe and had encouraging initial activity in patients with metastatic castration-resistant PCa.	R. Aggarwalet al. 2023 (111)
Pembrolizumab	Phase 2	mCRPC/529	laparib/a next-generation hormonal agent (NHA)	There was no significant improvement in rPFS or OS in pembrolizumab combined with laparib mCRPC patients compared with NHA.	E. S. Antonarakis et al., 2023 (112)
Pembrolizumab	Phase 2	mCRPC/14	HER2 bispecific antibody (HER2Bi)-armed activated T cells (HER2 BAT)	The combination has good safety and efficacy in the treatment of mCRPC.	U. N. Vaishampayanet al. 2023 (113)
Pembrolizumab	Phase 1/2	mCRPC/104	olaparib	Pembrolizumab in combination with laparib has shown antitumor activity and expected safety in patients with metastatic castration-resistant PCa.	E. Y. Yu et al., 2023 (114)
Nivolumab	Phase 1/2	mCRPC/6	high-dose-rate (HDR) brachytherapy	The combination of nivolumab with ADT and HDR was well tolerated and was associated with increased immune invasion and anti-tumor activity.	Z. Yuanet al. 2021 (115)
Nivolumab	Phase 2	mCRPC/84	docetaxel	Nivolumab combined with docetaxel is clinically active in chemotherapy-naïve mCRPC patients.	K. Fizazi et al., 2022 (116)
Nivolumab	Phase 2	mCRPC/-	Rucaparib	Nibulimab in combination with Lucaparib is effective in chemotherapy-naïve mCRPC patients, especially those with the BRCA1/2 mutation.	K. Fizazi et al., 2022 (117)
Nivolumab	Phase 2	mCRPCs/19	pTVG-HP vaccine	pTVG-HP vaccination combined with Nivolumab was safe and immunologically active, extending the time of disease progression but not eradicating the disease.	D. G. McNeel et al., 2023 (69)
Nivolumab	Phase 2	mCRPC/45	bipolar androgen therapy (BAT)	BAT may enhance the anti-tumor immune response, and immune checkpoint blocking further enhances this immune response.	M. C. Markowski et al., 2024 (118)

#### 4.2.1 CTLA4 inhibitors

Ipilimumab is a monoclonal antibody targeting CTLA-4, which can effectively block the binding of CTLA-4 to its ligand and inhibit the tumor-killing effect of T cells. E. D. Kwon et al. (100) examined the efficacy of ipilimumab following radiotherapy in individuals with advanced CRPC who experienced progression following docetaxel treatment (NCT00861614). A total of 799 patients were assigned in a random manner, with 399 receiving ipilimumab and 400 receiving a placebo. Following a brief monitoring period, the ipilimumab group did not show a significant difference in median OS compared to the placebo group (11.2 months vs. 10.0 months, *P*= 0.053). Immune-related adverse events were the most frequent grade 3-4 occurrences, with 101 patients (26%) experiencing them in the ipilimumab group compared to 11 patients (3%) in the placebo group. The toxic effects of the study drug resulted in four deaths (1%), all of which were in the ipilimumab group. Preliminary analyses showed no significant difference in OS between the ipilimumab and placebo groups. Subsequently, Fizazi et al. (102) conducted a comprehensive study on this group over an extended period of time. The ipilimumab group had a higher overall survival rate compared to the placebo group after 2 years (25.2% vs 16.6%) and 3 years (15.3% vs 7.9%). Comparing the rates at four years (10.1% vs 3.3%) and five years (7.9% vs 2.7%). The primary cause of death in seven patients (1.8%) in the ipilimumab group and one patient (0.3%) in the placebo group was reported to be drug toxicity during the study. After an extended period of observation, individuals who received ipilimumab exhibited improved survival outcomes, showing a 2-3 fold increase in OS rates at 3 years and beyond. Furthermore, T. M. Beer et al (101) evaluated the efficacy of ipilimumab in the treatment of mCRPC at the initial stage of chemotherapy without visceral metastasis. Patients in this Phase III trial were randomly allocated to receive either ipilimumab or a placebo in multiple centers. The study included 399 patients who were administered ipilimumab and 199 patients who were given a placebo. The results revealed that there was no significant difference in the short-term median OS between the group receiving ipilimumab and the control group (28.7 months vs 29.7 months, *P*=0.3667). However, the median PFS in the ipilimumab group was found to be significantly higher than in the placebo group (5.6 months vs 3.8 months, *P* < 0.05). In the ipilimumab group, 9 patients (2%) died due to treatment-related adverse events. No fatalities were reported in the control group. Grade 3-4 adverse events associated with immunity were observed in 31% and 2% of patients, respectively. Research indicates that ipilimumab may enhance OS in the short term for individuals with mCRPC, while also potentially increasing long-term survival rates. However, the incidence of treatment-related adverse events is higher.

Previous studies have not shown a significant benefit of Ipilimumab monotherapy in the treatment of mCRPC. Researchers have explored the potential synergistic effects of combining CTLA-4 and PD-1 monoclonal antibodies for the treatment of mCRPC. P. Sharma et al. (103) reported the largest trial of ipilimumab combined with nivolumab for mCRPC treatment (NCT02985957). The cohort had a median follow-up time of 11.9-13.5 months with 45 participants, and achieved an objective response rate of 10%-25%. Metastatic PCa that expresses the androgen receptor variant 7 (AR-V7) is associated with a poor prognosis, limited therapeutic options, and reduced survival rates. E. Shenderov et al. (104) combined ICIs in the treatment of AR-V7-expressed mCRPC. This study was a cohort, non-randomized phase 2 study. Ipilimumab and nivolumab were administered to a total of thirty patients. The study results indicated that the combination of nivolumab and ipilimumab had limited effectiveness in PCa patients with AR-V7 expression, leading to a decision not to pursue further investigation. Therefore, the combination of ICIs in the treatment of mCRPC has not achieved significant efficacy, which may be due to the complex immunosuppressive microenvironment of PCa.

Ipilimumab in combination with the sipuleucel-T strategy for mCRPC was also evaluated. Investigators explored whether administration of ipilimumab following sipuleucel-T treatment could alter immune and/or clinical response to this treatment (63). A total of 50 mCRPC patients enrolled in this clinical trial (NCT01804465) received ipilimumab immediately after completion of sipleul-T or a 3-week delay. The research discovered that the mixture was easily endured without any unforeseen negative occurrences. Clinical responses were observed in 6 of the 50 patients, 3 of which lasted longer than 3 months. There was no significant correlation between the length of time ipilimumab was used and the clinical response or toxicity. The use of multiple treatments did lead to the activation of CD4 and CD8 T cells, especially in the early treatment schedule. Similarly, C. Jochems et al. (99) reported the clinical results of a Phase I trial that combined ipilimumab with a PROSTVAC vaccine in patients with mCRPC. Ipilimumab and PROSTVAC vaccine were administered to a total of thirty patients. PSA levels decreased in 58% of the 24 patients who had not received chemotherapy before. The concurrent therapy did not worsen immune-related side effects linked to ipilimumab. The middle OS duration was 2.63 years, ranging from 1.77 to 3.45 years. This confirms that the use of ICIs in combination with cancer vaccines has the potential to improve patient outcomes. However, larger clinical trials of immunotherapy are needed for further evaluation.

#### 4.2.2 PD-1/PD-L1 inhibitors

##### 4.2.2.1 Pembrolizumab

Pembrolizumab, a monoclonal antibody of the IgG4 class, blocks the PD-1 receptor to treat cancer through immunotherapy. As a PD-1 inhibitor, it has shown obvious antitumor efficacy in several solid tumors. Pembrolizumab has shown antitumor activity against PD-L1-positive mCRPC. J. N. Graff et al. (106) reported surprising effectiveness against tumors in patients with mCRPC who were given pembrolizumab. Patients were administered pembrolizumab, resulting in a rapid decrease in PSA to ≤ 0.2 ng/ml for three out of the initial 10 patients. Two of the three responders had baseline tumor biopsies. Immunohistochemistry showed leukocyte infiltration and PD-L1 expression of CD3+, CD8+ and CD163+. This study suggests that pembrolizumab applied to mCRPC can induce a favorable tumor-specific immune response and inhibit tumor progression. Similarly, E. S. Antonarakis et al. (107) evaluated the antitumor activity and safety of pembrolizumab in mCRPC, and obtained good curative effect. In the KEYNOTE-199 Phase II trial, three cohorts of patients with mCRPC were treated with docetaxel along with one or multiple targeted hormonal treatments. All patients received pembrolizumab every 3 weeks for a total of 35 cycles. A total of 258 patients were included. Disease control rates ranged from 10%-22%, and median survival ranged from 7.9-14.1 months. In patients with bone-dominant mCRPC who had prior treatment with doxetaxel and targeted endocrine therapy for solid tumors, Pembrolizumab alone demonstrated effectiveness against tumors and was deemed safe, according to the research. The observed response appears to be persistent, and OS estimates are encouraging. The surprising and powerful response seen in the above study should be re-examined for PD-1 inhibition of PCa.

A study demonstrated that patients with PCa who showed resistance to the novel androgen receptor inhibitor enzalutamide displayed elevated levels of PD-L1 expression on circulating immune cells (119). The researchers theorized that introducing PD-1 inhibition to these individuals may trigger a significant cancer reaction. In a Phase II study conducted by J. N. Graff et al. (108), the combination of enzalutamide and pembrolizumab was assessed in a cohort of 28 patients with mCRPC. In 5 of the 28 patients (18%), PSA decreased by 50% or more. Three of the 12 patients (25%) with measurable disease at baseline achieved an objective response. Median OS was 21.9 months for all patients (95% CI: 14.7 to 28.4 months) and 41.7 months for responders. This study showed that pembrolizumab was active in mCRPC when enzalutamide was added. The response is deep and long-lasting, and does not require defects in tumor PD-L1 expression or DNA repair. Besides, A. E. Ross et al. (109) evaluated the safety and feasibility of combination pembrolizumab and androgen deprivation in the treatment of metastatic hormone-sensitive PCa. The study included 12 patients with newly diagnosed minimally metastatic PCa. The individual received a complete prostate cryoablation along with temporary ADT and pembrolizumab. The findings indicated that 42% (5 out of 12) of individuals exhibited PSA levels below 0.6 ng/mL after one year, with only 2 of them showing normalized testosterone levels at that time. The median duration of PFS was 14 months, while the median duration of survival without systemic therapy was 17.5 months. Immunohistochemistry did not detect PD-L1 expression in patients with assessable tissue. Patients with metastatic PCa can tolerate total prostate cryoablation along with short-term androgen deprivation and pembrolizumab without any safety issues. While the localized disease appears to be treated effectively in most men, the regimen only leads to sustained disease control in a few cases after testosterone recovery.

In the clinical setting, docetaxel is frequently selected for patients with mCRPC who have become resistant to abiraterone or enzalutamide and need a more potent therapy. E. Y. Yu et al. (110) assessed the effectiveness and safety of pembrolizumab in combination with docetaxel for treating patients with mCRPC. Among the 104 treated patients, the confirmed PSA response rate was 34% and the confirmed ORR was 23%. Median radiologic progression-free survival (rPFS) and OS were 8.5 months and 20.2 months, respectively. The combination demonstrated a manageable safety profile in this patient population. The combination of pembrolizumab and docetaxel demonstrated effectiveness against tumors in mCRPC patients who had not received chemotherapy and were being treated with abiraterone or enzalutamide. To determine whether pembrolizumab therapy after treatment with (177) Lu-Prostate-specific membrane antigen (PSMA)-617 is safe and induces lasting clinical benefit, R. Aggarwal et al. (111) conducted a Phase 1 study. An objective response was confirmed in 25 of the 43 patients included. Two of the 43 patients (5%) had grade 3 or more severe treatment-related adverse events. After pembrolizumab maintenance therapy, a sole administration of (177) Lu-PSMA-617 was well-tolerated and showed promising early effects in individuals with metastatic castration-resistant PCa. The above study suggests the potential value of pembrolizumab in combination with chemoradiotherapy for mCRPC treatment, and more randomized controlled studies are needed to confirm their efficacy.

Pembrolizumab and olaparib showed single-agent activity in previously treated mCRPC patients. E. Y. Yu et al. (114) evaluated the effectiveness and safety of combining pembrolizumab with olaparib for treating mCRPC. The study ended with 102 patients receiving treatment. The average duration from initial administration to the end of data collection was 24 months. The effective rate of diagnosed PSA was 15%. Among patients with detectable disease, the verified overall response rate was 8.5%, with 5 cases of partial remission. The median PFS rate was 4.5 months (95%CI: 4.0-6.5), while the median OS was 14 months (95%CI: 10.4-18.2). Consistency was observed in the clinical activity of subgroups with PD-L1 positivity and mutations in homologous recombination repair. Ninety-three patients (91%) experienced adverse events associated with treatment. The research indicated that the safety profile of pomerzumab combined with olaparib was similar to that of a standalone treatment and exhibited anti-cancer effects in mCRPC patients who had received prior docetaxel treatment without molecular selection. Similarly, E. S. Antonarakis et al. (112) evaluated the efficacy of pembrolizumab in combination with olaparib and a new generation hormone agent (NHA) for mCRPC without selected biomarkers in a Phase III clinical study. Subjects were assigned randomly (2:1) to receive either pembrolizumab combined with olaparib or NHA (abiraterone or enzalutamide). Pembrolizumab + olaparib was given to 529 participants through random assignment, while 264 participants received NHA. In the final rPFS and OS analysis, there was no difference between the two groups. This study showed that pembrolizumab combined with olaparib did not significantly improve rPFS or OS in heavily pretreated mCRPC patients with no biomarker selection compared to NHA. Besides, U. N. Vaishampayan et al. (113) conducted a Phase II study to evaluate the safety and efficacy of the combination of HER2 bispecific antibodies (HER2Bi) armed activated T cells (HER2 BAT) and pembrolizumab. Out of the total of 14 patients, five achieved the main goal of PFS at the 6-month mark, representing 38.5% (95%CI: 19.5% - 76.5%). The middle PFS was 5 months with a median survival of 31.6 months. The safety and good efficacy of the combination deserve further study.

Pembrolizumab in combination with the cancer vaccine is also being tested for mCRPC with promising results. D. G. McNeel et al. (64) reported a trial of MVI-816 administered simultaneously or sequentially to pembrolizumab over 12 weeks in patients with mCRPC. Of the 25 patients with measurable disease, a partial response was confirmed in 1 patient with microsatellite instability tumors. In 4/40(10%) patients, PSA decreased by >50%. The overall radiological PFS rate at 6 months was 47.2%. Thirty-two percent of patients did not progress in the trial beyond six months. The average survival time was 22.9 months with a confidence interval of 95% ranging from 16.2 to 25.6 months. The occurrence of immune-related adverse events was significantly associated with longer treatment duration (HR=0.42, *P*=0.003). The results of this study suggest that the combination of programmed cell death 1 blocking with MVI-816 is safe, increases tumor-specific T cells, and can lead to favorable 6-month disease control rates.

##### 4.2.2.2 Nivolumab

In recent years, nivolumab, a monoclonal antibody of the human immunoglobulin G4 class, has been utilized in tumor immunotherapy by binding to the PD-1 receptor, disrupting its interaction with PD-L1 and PD-L2, and inhibiting the immunosuppressive effects of the PD-1 pathway. K. Fizazi et al. (116) investigated docetaxel combined with nivolumab in the treatment of mCRPC in a non-randomized, multi-cohort phase II trial (NCT03338790). The study included eighty-four patients with mCRPC who were starting chemotherapy. The ORR was confirmed at 40.0% (95% CI: 25.7-55.7) and the PSA response rate was confirmed at 46.9% (95% CI: 35.7-58.3). The median rPFS and OS were 9.0 months (95% CI: 8.0-11.6) and 18.2 months (95%CI: 14.6-20.7), respectively. Nivolumab in combination with docetaxel is clinically active in chemotherapy-naive mCRPC patients. In addition, nivolumab combined with rucaparib was found to be effective in patients with homologous recombination defect positive post-chemotherapy or chemotherapy-naive mCRPC, especially those with BRCA1/2 mutations (117). Similarly, in a study conducted by Z. Yuan et al. (115), a Phase I/II trial was undertaken to assess the safety and potential synergistic effects of combining brachytherapy with immunotherapy for PCa. The study aimed to investigate the feasibility, safety, and benefits of administering nivolumab in conjunction with high dose rate (HDR) brachytherapy and androgen deprivation therapy (ADT) in patients with PCa. A Phase I trial included six patients who were monitored for a minimum of 3 months following administration of Nivolumab. Overall, the combination of Nivolumab with ADT and HDR therapy was well received by patients. At one month after receiving four cycles of nivolumab and HDR brachytherapy, three patients (50%) exhibited an initial positive reaction, with no remaining tumors found. In early responders, there was an increase in CD8+ and FOXP3+/CD4+ T cells in tissues, while CD4+ effector T cells were elevated in the peripheral blood. The combination of Nivolumab, ADT, and HDR was well received and led to enhanced immune infiltration and anti-cancer effects.

Bipolar androgen therapy (BAT), a form of high-dose testosterone treatment given at intervals, is used as a treatment approach for patients with mCRPC. More recently, M. C. Markowski et al. (118) reported the results of a multicenter, single-arm Phase 2 study (NCT03554317) that included 45 heavily pretreated mCRPC patients. The patients received nivolumab following 3 cycles of BAT monotherapy. Following a typical observation period of 17.9 months, the median time to recurrence or progression was 5.6 months (95% CI 5.4-6.8) and the median OS was 24.4 months (95% CI 17.6-31.1). The study findings indicated that BAT/nivolumab was well received, with only five (11%) severe adverse events related to the drug. The data indicates that BAT could boost the immune response against tumors, especially when combined with ICIs. Furthermore, Neel et al. (69) evaluated the efficacy of nivolumab combined with pTVG-HP in patients with early recurrent PCa. PSA levels decreased by more than 50% in four out of 19 patients, representing 21% of the total. The median PSA doubling time was 5.9 months prior to treatment, increased to 25.6 months post-treatment (*P*=0.001), and then decreased to 9.0 months one year after treatment. The research validated the safety and immune-boosting effects of combining nivolumab with pTVG-HP vaccine, leading to a longer period before disease progression.

##### 4.2.2.3 Avelumab

*Avelumab, a PD-L1 antibody that is fully humanized, effectively inhibits the interaction between PD-L1 and PD-1, thus boosting the ability of T cells to kill tumors by counteracting the immunosuppressive effects of the TME.* A. Rodriguez-Vida et al. (105) investigated the safety and efficacy of avelumab plus carboplatin in A single-arm Phase Ib study of mCRPC. The study consisted of 26 patients in total. 7.7% of patients had PSA response rate≥50%. The objective effective rate was 17.6%, and the complete effective rate was 1 case (5.9%). The middle radiological PFS was 6.6 months (95% CI: 4.28 and 9.01), while the middle OS was 10.6 months (95% CI: 6.68 and not reached). 73% of grade 3-4 adverse events related to treatment. The research showed that the combination of avelumab and carboplatin was safe and led to extended OS in a group of patients who had undergone extensive prior treatments. In addition, E. M. Kwan et al. (120) evaluated the effectiveness and safety of avelumab, when used alongside stereotactic ablative radiotherapy for managing mCRPC. This prospective Phase 2 study enrolled 31 patients with progressive mCRPC who had received at least one previous androgen receptor-directed therapy. The median follow-up was 18.0 months. The rate of disease control was 48% (95% CI: 30-67%), while the overall response rate was 31% with a confidence interval of 11-59%. The ORR of unirradiated lesions was 33% (95% CI: 10-65%). The median PFS rate was 8.4 months with a confidence interval of 95% from 4.5 to not reached, while the median OS rate was 14.1 months with a confidence interval of 95% from 8.9 to not reached. These studies indicate that Avelumab in combination with other oncology therapies has great potential in mCRPC, and more clinical studies are needed to confirm their efficacy and safety.

## 5 CAR-T cells in the treatment of PCa

### 5.1 CAR-T cells technology

While advancements have been made in the treatment of blood cancers, the efficacy of CAR-T technology in addressing solid tumor treatments remains limited. Hematologic malignancies commonly exhibit consistent target antigens across the majority of tumor cells, in contrast to solid tumors which display heterogeneous tumor antigens that can vary both within the same tumor location and between primary tumors and metastatic sites. It is difficult to select the ideal target antigen of solid tumor, which directly limits the specificity and effectiveness of CAR-T cells. Furthermore, the TME within solid tumors create a barrier that hinders the infiltration of CAR-T cells and the recognition of tumor antigens. Furthermore, numerous challenges remain for CAR-T cells to effectively penetrate tumor tissue and induce immune responses. On the one hand, metabolic disorders, including oxidative stress in TME, nutrient deprivation, hypoxia, and abnormal metabolic accumulation of acidic pH and metabolites, etc. Moreover, there are also factors that suppress the immune system, like molecules that suppress the immune response, such as TGF-β, IL-10, and other cytokines (121). Ultimately, the intrinsic inhibitory processes within T cells, including the enhancement of immune-suppressing receptors (PD-1, LAG-3, and TIM-3) or molecules, result in the suppression of T cell stimulation and potential functional depletion (122). These factors contribute to the limited success of CAR-T cell therapy in treating solid tumors and the lack of positive results in clinical trials.

**Figure 4:**
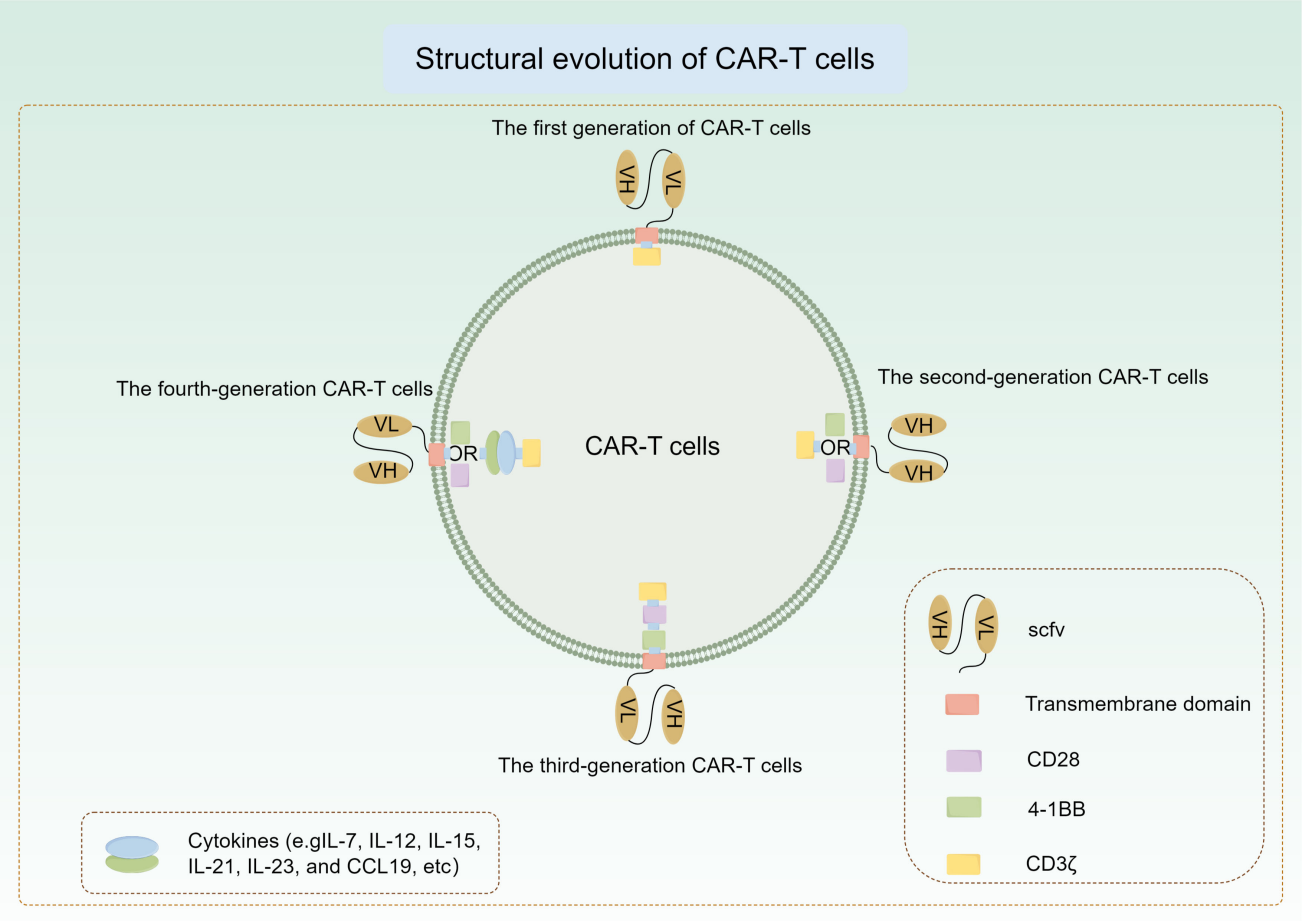
Demonstration of structural optimization during CAR-T cell development by Figdraw.

The first generation of CAR-T relies on CD3ζ to mediate the activation of T cells. This kind of CAR-T lacks intracellular costimulatory signal and cannot provide long-term T cell expansion signal, so the clinical efficacy is limited (123). The second-generation CAR-T cells were created by incorporating the co-stimulatory molecule CD28 into the intracellular domain of the initial CAR-T cells, resulting in a notable rise in the production of IL-2 and other cytokines when compared to the first-generation CAR-T cells (124). After that, the researchers added a co-stimulatory molecule(4-1BB) to the second generation to produce the third generation of CAR-T cells. Research has shown that the expansion, extended viability, cytokine release, and elimination of tumors by advanced CAR-T cells containing extra co-stimulatory regions are greatly enhanced following antigen activation (125). In recent years, in order to deal with the problems encountered in CAR-T therapy, researchers have made a series of modifications to its structure. Specific cytokine gene fragments are inserted into the intracellular region of CAR-T cells to enable the production and release of particular cytokines upon activation. This process aims to boost T cell proliferation and activation, enhance non-specific anti-tumor immune responses, promote CAR-T cell infiltration into tumors, and ultimately enhance treatment outcomes. Cytokines like IL-7, IL-12, IL-15, IL-21, IL-23, and CCL19 are examples of these molecules (126). These CAR-T cells are also known as fourth-generation CAR-T cells (**Figure 4**). Continuous enhancements in structural optimization and functional modifications have increased the effectiveness and safety of CAR-T cells, making the tailored design of CAR-T cells for various types of tumors a current focus of research.

### 5.2 Potential targets and related research

The notable efficacy of CAR-T cells in preclinical studies targeting various antigens has prompted an increase in clinical trials aimed at treating PCa (**Table 3**). The clinical trials published so far on CAR-T cells targeting PSMA and PSCA for PCa treatment have primarily concentrated on these specific targets, but have not achieved significant advancements (127). At present, the main problems are the inhibition effect of PCa microenvironment on CAR-T cells and the lack of specific targets. To address this dilemma, researchers try to optimize the function of CAR-T cells by using gene modification strategies, in order to achieve significant success in the treatment of PCa. Furthermore, researchers are currently exploring the development of novel targets using proteomics technology to keep pace with the swift integration of CAR-T cells in the treatment of PCa. Among them, STEAP1, STEAP2 and F77 have been used as new targets for CAR-T therapy of PCa, and clinical studies of CAR-T cells targeting STEAP1 and STEAP2 have also begun.

**Table 3:** Different CAR-T cells clinical trials.

Interventions	Conditions	Targeted antigen	Phase	Enrollment (n)	NCT Number	Status	First Posted	Sponsor
PSMA-CAR-T Cells	mCRPC	PSMA	Phase 1	60	NCT04249947	Active, not recruiting	2020-01-31	Poseida Therapeutics, Inc.
PSMA-targeted CAR-T Cells	CRPC	PSMA	Phase 1	20	NCT05354375	Recruiting	2022-04-29	The Affiliated Hospital of Xuzhou Medical University
TmPSMA-02 CAR-T Cells	mCRPC	PSMA	Phase 1	18	NCT06046040	Recruiting	2023-09-21	University of Pennsylvania
Enhanced autologous PSMA-CAR-T	mCRPC	PSMA	Phase 1	18	NCT06228404	Recruiting	2024-01-29	Shanghai Changzheng Hospital
PD-1 Silent PSMA/PSCA Targeted CAR-T	PCa	PSMA/PSCA	Phase 1	12	NCT05732948	Recruiting	2023-02-17	Shanghai Unicar-Therapy Bio-medicine Technology Co.,Ltd
PSCA-Targeting CAR-T Cells	mCRPC	PSCA	Phase 1	21	NCT05805371	Recruiting	2023-04-10	City of Hope Medical Center
PSCA-CAR-T Cells	mCRPC	PSCA	Phase 1	14	NCT03873805	Active, not recruiting	2019-03-13	City of Hope Medical Center
PSCA-Targeted CAR-T Cells (BPX-60	mCRPC	PSCA	Phase 1Phase 2	151	NCT02744287	Suspended	2016-04-20	Bellicum Pharmaceuticals
L1CAM-Targeted CAR-T	Locally Advanced and Unresectable or Metastatic Small Cell Neuroendocrine PCa	L1CAM	Phase 1	20	NCT06094842	Not yet recruiting	2023-10-23	Fred Hutchinson Cancer Center
PSMA-targeted CAR-T Cells	mCRPC	PSMA	Phase 1	13	NCT01140373	Active, not recruiting	2010-06-09	Memorial Sloan Kettering Cancer Center
PSMA-specific CAR-T Cell	PCa	PSMA	Phase 1 Phase 2	100	NCT04429451	Recruiting	2020-06-12	Shenzhen Geno-Immune Medical Institute
TmPSMA-02 CAR-T Cells	mCRPC	PSMA	Phase 1Phase 2	1	NCT05489991	Terminated	2022-08-05	Tceleron Therapeutics, Inc.
Steap2 -targeted CAR-T Cells	Metastatic PCa	Steap2	Phase 1 Phase 2	60	NCT06267729	Recruiting	2024-02-20	AstraZeneca
CART-PSMA-TGFβRDN cells	mCRPC	PSMA	Phase 1	19	NCT03089203	Recruiting	2017-03-24	University of Pennsylvania
Anti-STEAP1 CAR-T-cells	Castration-Resistant Prostate Carcinoma Metastatic Prostate Adenocarcinoma	STEAP1	Phase 1 Phase 2	48	NCT06236139	Not yet recruiting	2024-02-01	Fred Hutchinson Cancer Center
CART-PSMA-TGFβRDN	mCRPC	PSMA	Phase 1	16	NCT04227275	Terminated	2020-01-13	Tceleron Therapeutics, Inc.
PD1-PSMA-CART cells	CRPC	PSMA	Phase 1	3	NCT04768608	Completed	2021-02-24	Zhejiang University

#### 5.2.1 PSCA

PSCA, a protein found on the surface of prostate epithelial cells, as well as in primary and metastatic PCa cells, presents a potential target for immunotherapeutic interventions in PCa. V. Hillerdal et al. (128) developed a third generation CAR targeting PSCA, including the CD28, OX-40, and CD3ζ signal domains. It was confirmed that PSCA-CAR-T cells exhibited targeted secretion of IFN-γ and IL-2. Furthermore, PSCA-CAR-T cells efficiently eliminated cancer cells that displayed PSCA in a laboratory setting, and the use of PSCA-CAR-T cells notably slowed the progression of tumors implanted under the skin and extended the lifespan of mice. The PD-1/PD-L1 pathway is widely recognized as a crucial mechanism used by cancer cells to evade destruction by CAR-T cells through immunosuppression. J. E. Zhou et al. (129) developed third-generation PSCA-CAR-T cells with PD-1 suppression through shRNA gene silencing, aiming to boost CAR-T cells’ ability to fight tumors by inhibiting the PD-1/PD-L1 pathway. PD-1-silenced PSCA-CAR-T cells exhibited superior cytotoxicity and cytokine secretion compared to regular CAR-T cells at an effector-to-target cell ratio of 8:1. Within one week, tumor volume was significantly decreased in tumor models that received treatment with PSCA-CAR-T cells with PD-1 silenced. The research demonstrates that using shRNA to silence PD-1 is a successful approach in inhibiting the PD-1/PD-L1 immune suppression pathway and improving the effectiveness of CAR-T cells in subcutaneous xenograft treatment. Current adjuvant treatment strategies for bone mCRPC have been largely unsuccessful. J. S. Frieling et al. (130) investigated the efficacy of PSCA-CAR-T cells in the treatment of bone mCRPC. PSCA-CAR-T cells in preclinical mouse models of bone mCRPC led to quick and substantial tumor regression, as well as improved survival and decreased bone disease associated with cancer. The data provided evidence for the effectiveness of PCA-CAR-T cell therapy in managing mCRPC.

#### 5.2.2 PSMA

PSMA, a transmembrane glycoprotein found on the cell membrane, is highly expressed in PCa. As a molecular target in PCa, it has been extensively utilized and researched for the last twenty years. Similarly, it is a potential target for the application of CAR-T cell technology in PCa treatment. J. Alzubi et al. (131) designed a CAR that recognizes PSMA and applied this CAR-T cell to a preclinical PCa model. PSMA CAR-T cells were administered via local injection in a preclinical mouse model, leading to the eradication of established human PCa xenografts *in vivo*. Moreover, the co-administration of systemic intravenous CAR-T cells with non-ablative low-dose docetaxel chemotherapy demonstrated significant efficacy in inhibiting tumor growth, the outcome that was not observed with either docetaxel or CAR-T cells as standalone treatments. To enhance the performance of PSMA CAR-T cells, D. Wang and colleagues developed a group of PSMA CAR-T cells that were genetically altered to include IL23 (IL23-PSMA-CAR-T) and examined their performance in a mouse experiment (132).During the co-culture trial, the IL23-PSMA-CAR-T cells exhibited a notably greater proliferation rate compared to the control CAR-T cells. IL23-PSMA-CAR-T cells exhibited a markedly elevated production of cytokines compared to the other CAR-T cell groups. IL23-PSMA-CAR-T cells demonstrated superior functionality in NSG mouse models compared to other CAR-T cell groups, leading to tumor eradication starting on day 14 post T cell infusion, with immediate weight recovery observed. There was a notable increase in CD45RO+ CD8+ T cells and CD127+ CD4+ CAR-T cells in IL23-PSMA-CAR-T mice. The results indicate that the role of PSMA-CAR-T modified with IL-23 has great potential in the eradication of PCa.

CAR-T cells face difficulties in solid tumors due to the presence of immunosuppressive TME that contain elevated amounts of various inhibitors, such as TGF-β, T cells immunoglobulin, and mucin domain 3 (TIM3). L. Tang et al. (133) constructed PSMA-CAR-T cells targeting both TIM3 and TGFβ in order to improve their tumor-killing ability. *In vitro* and animal experiments were conducted to assess the cytotoxicity of TIM3/TGF-PSMA-CAR-T cells. Exogenous TGF-β and TIM3-activating antibodies were discovered, leading to the successful elimination of PSMA-positive PCa cells by TIM3/TGF-PSMA-CAR-T cells. Furthermore, when transplanted into immunodeficient mouse tumor models *in vitro*, these cells demonstrated the capacity to eradicate tumor tissue and extend survival without causing notable toxic reactions. Similarly, V. Narayan et al. (134) reported the results of a phase 1 clinical trial of PSMA-CAR-T cells with TGF-β receptor targeting function (NCT03089203) for PCa treatment. Thirteen patients were eventually treated with TGF-β PSMA-CAR-T cells. In one patient, there was significant expansion of cloned CAR-T cells and a >98% reduction in PSA. In the other three patients, PSA decreased by≥30% and CAR-T cell failure was accompanied by the upregulation of multiple TME location-inhibiting molecules after adoptive cell metastasis. Research indicates that it is possible and promising to use gene editing techniques to enhance the ability of PSMA-CAR-T cells to destroy tumors in PCa.

#### 5.2.3 NKG2D

NKG2D ligand is a receptor that activates NK cells and is predominantly found on numerous cancer cells, such as PCa, while typically being absent or expressed minimally in normal tissues (135, 136). Numerous CAR utilizing NKG2D have been created and researched for their diverse therapeutic benefits in combating different types of tumors (137). C. He et al. (138) developed NKG2D-CAR-T cells that co-express IL-7 and utilized them in the treatment of PCa to enhance therapeutic efficacy by leveraging the co-expression of IL-7. The findings indicated that NKG2D-CAR-T cells exhibited markedly enhanced cytotoxicity against PCa in both laboratory and animal studies compared to T cells that had not been genetically modified. Furthermore, IL7-NKG2D-CAR-T cells exhibited superior tumor-fighting capabilities and effectively suppressed tumor growth in xenograft models. The findings suggest that targeting NKG2D with CAR-T cells holds promise for treating PCa, and the inclusion of IL-7 may enhance the effectiveness of NKG2D-based CAR-T cell therapy, offering a novel approach for adoptive cell therapy in PCa.

#### 5.2.4 B7-H3

B7-H3, an immune-suppressing molecule, is found at increased levels in various cancer types such as pancreatic ductal adenocarcinoma, ovarian cancer, lung cancer, clear cell kidney cancer, and PCa (139, 140). Elevated B7-H3 expression levels in PCa are associated with high gleason scores, advanced stages, metastases, and poor patient outcomes (141). Crucially, B7-H3 is expressed very little in healthy tissues (142). New research has uncovered compelling proof that B7-H3 controls immune responses mediated by T cells and that blocking B7-H3 could result in the proliferation of T cells (142). S. Li et al. (143) developed CAR-T cells specifically targeting B7-H3 to explore their tumor-killing potential against PCa *in vitro* and *in vivo*. B7-H3 CAR-T cells efficiently suppress PCa growth through antigen-specific mechanisms. Furthermore, tumor cells have the ability to stimulate the growth of CAR-T cells in a laboratory setting and produce elevated amounts of IFN-γ and TNF-α cytokines. The findings suggest that B7-H3 could be a promising focus for treating PCa, backing the advancement of B7-H3-targeted CAR-T cells for PCa treatment. New research indicates that radiation treatment (RT) increases the levels of B7-H3 in PCa stem cells (PCSCs). Y. Zhang et al. (144) investigated whether B7-H3-CAR-T cells could target anti-RT PCSCs *in vitro* and *in vivo*. B7-H3 expression was discovered to be higher on PCSCs compared to PCa cells, resulting in B7-H3 CAR-T cells exhibiting greater cytotoxicity towards PCSCs than PCa cells. Furthermore, RT markedly increased the levels of B7-H3 in both PCSCs and PCa cell+s. The RT and B7-H3 CAR-T cells combination proved to be more potent in suppressing the growth of hormone-insensitive PCa xenografts in immunodeficient mice compared to using RT or CAR-T cells individually. The above studies indicate that utilizing CAR-T cells directed at B7-H3 could serve as a valuable supplementary treatment for late-stage PCa.

#### 5.2.5 STEAP1/2

Prostatic six transmembrane epithelial antigen 1 (STEAP1) was first identified more than 20 years ago and is thought to be highly expressed in PCa. In more than 80% of mCRPC cases invading the bone or lymph nodes, as well as in numerous other types of cancer, there is a high level of expression of STEAP1 (145). STEAP1 belongs to the STEAP family of metal reductases and can form auto trimers or heterotrimers with other STEAP proteins (146). The functional role of STEAP1 in enhancing cancer cell proliferation, invasion, and epithelial-to-mesenchymal cell transformation is well established (147–149). The limited expression of STEAP1 in normal tissues makes it a very attractive target for cancer therapy. STEAP1 is a cell surface antigen used in the targeted therapy of PCa. CAR-T cells that target STEAP1 demonstrated responsiveness even at low levels of antigen and exhibited effectiveness against tumors in models of metastatic PCa (150). Besides, the large-scale genomics and proteomics research have identified prostatic six transmembrane epithelial antigen 2 (STEAP2) also can be used as a superior PCa treatment target antigen (151, 152). Consistent with this finding, the study showed that STEAP2 is richly expressed in all stages of PCa and can be used as a prognostic biomarker due to its association with the gleason score (153–155). P. Zanvit et al. (156) prepared STEAP2-targeting CAR-T cells (AZD0754) for the treatment of PCa. AZD0754 showed potential effectiveness in mouse models with patient-derived xenografts expressing STEAP2, along with promising preclinical safety results. The data highlight the value of STEAP1 and STEAP2 in the treatment of PCa and their potential application as adjunctive therapies for advanced PCa.

#### 5.2.6 F77

F77, a distinctive carbohydrate antigen found on both androgen-dependent and androgen-independent PCa cells, could serve as a promising target for immunotherapy. Immunohistological research indicated the absence of F77 expression in healthy colon, kidney, cervix, pancreas, lung, skin, and bladder tissues, validating the restricted overexpression of the F77 antigen in PCa (157). P. Grover et al. (158) constructed F77 targeting CAR-T cells with CD28 and 4-1BB co-stimulatory signals. F77-CAR-T cells eliminate tumor cells by releasing cytokines in a manner that relies on F77 expression. The F77-CAR-T cells specifically targeted and eliminated PCa in a human xenograft model with PC3 cells. The results validate F77 as a potential target for immunotherapy in PCa and other cancers characterized by this unusual carbohydrate pattern.

## 6 The dilemma and promise of PCa immunotherapy

In recent years, immunotherapy has significantly altered prevailing perspectives on cancer treatment and fostered a profound comprehension of tumor immunotherapy among the general populace. Cancer vaccines, ICIs, and CAR-T therapy function by stimulating the body’s adaptive immune response to recognize tumor antigens, leading to the eradication of malignant cells and the potential for tumor remission. Numerous basic and clinical studies have been undertaken to investigate the efficacy of the aforementioned strategies in the treatment of PCa, resulting in notable advancements. Cancer vaccines represented by sipuleucel-T are approved by the US FDA for the treatment of asymptomatic or mild mCRPC (37). Secondly, ICIs combined with other therapeutic strategies have achieved initial efficacy in the treatment of mCRPC, which is manifested in effective disease control rate and better OS. In addition, CAR-T cells has shown good tumor-killing efficacy in preclinical studies of PCa. At present, more clinical studies have been carried out, and most of the reported research results have shown good tolerance, and found the tumor immune response induced by CAR-T cells. However, no complete cure for PCa has been found.

Immunotherapy strategies in the treatment of advanced PCa offer promise but also face difficulties. The treatment of solid tumors should not only target cancer cells, but also focus on TME. TME in solid tumors first constitutes a physical barrier, affecting the infiltration of immune cells and the recognition of tumor antigens. In addition, immune cells need to overcome immunosuppressive TME to exert tumor immune effect (117). Secondly, PCa is called “cold tumor” because of its weak immune response function and low TMB (94). This means that fewer immune cells enter the tumor tissue. This may be the reason why cancer vaccines and ICIs have not shown complete efficacy in most clinical studies of PCa treatment (39, 71, 100, 101). Due to the tumor heterogeneity of different individuals with solid tumors, immunotherapy strategies applied to PCa should select an adaptive patient population, and then optimize the dose of drugs to achieve the best clinical response rate. Therefore, tumor immunotherapy strategies need to pay more attention to the patient population to which they are adapted while optimizing function. In addition, the combination of tumor immunotherapy strategies and other therapeutic methods is also a direction of the adjuvant therapy of PCa, such as the combination of cancer vaccine and ICIs applied to the treatment of PCa (60, 63), and the research results also confirm that the combination therapy is beneficial. The above studies made a preliminary exploration of cancer vaccine combined with other therapeutic measures, and laid a theoretical foundation for the next widespread application of cancer vaccine in the treatment of PCa.

Furthermore, the choice of a TSAs is crucial in the implementation of tumor immunotherapy. CD19 is a specific membrane protein of B cells, so CAR-T cells targeting CD19 have been successful in the treatment of B-cell origin lymphoma and leukemia (159). CAR-T cell technology has not made a breakthrough in the treatment of solid tumors. One of the main problems is that the TSAs of tumors are not found. At present, the basic and clinical research targets used in the treatment of PCa are TAAs (PSCA, PSMA, NKG2D, B7-H3, etc) (160–166), which are also expressed in other normal tissues and organs. Similarly, this is the dilemma that limits the use of cancer vaccines for tumor treatment. Target antigens are expressed in normal tissues and organs, and the tumor immune response activated by immunotherapy strategies will inevitably damage normal tissues and organs. This will lead to the problem of side effects of immunotherapy, and most of the current clinical studies of solid tumor immunotherapy have shown a high incidence of side effects. Therefore, it is necessary to screen TSAs of PCa with the help of proteomics and transcriptomics to lay a foundation for the widespread application of immunotherapy in the treatment of PCa. CAR-T is also the future direction of PCa immunotherapy, and PCa is expected to become the first solid cancer to receive FDA approval for the use of CAR-T.

In addition, immunotherapy strategies can activate not only tumor-specific immune responses, but also adaptive immune responses. However, immunotherapy for tumors was originally developed to treat progressive PCa. The majority of the research focused on males with advanced PCa. Is it feasible to combine immunotherapy with surgery to achieve radical treatment in the early stages of the tumor? A. M. A. Tryggestad et al. (65) found DCs vaccine is of great value in reducing BCR in patients after robot-assisted laparoscopic prostatectomy. Therefore, immunotherapy strategies can be applied to the treatment of early and middle stage PCa to slow down the progression of PCa as much as possible. By further exploring the mechanism of immunotherapy strategies in the microenvironment of early PCa, it may be possible to find a breakthrough for the immunotherapy of advanced PCa.

In conclusion, tumor immunotherapy has been extensively explored in research on PCa treatment, encompassing cancer vaccines, ICIs, and CAR-T cells therapy. The therapeutic approaches employed vary from single-agent treatments to combination regimens, with certain studies demonstrating modest clinical efficacy, albeit insufficient for broad clinical application. Reversing the inhibitory TME and screening out meaningful targets play a decisive role in immunotherapy of PCa. With the widespread application of transcriptomics and proteomics in PCa screening, human understanding of the inhibitory TME is gradually deepening, and immunotherapy strategies will certainly bring extensive benefits to the treatment of PCa.
